# Positional Isomerism in the N^N Ligand: How Much Difference Does a Methyl Group Make in [Cu(P^P)(N^N)]^+^ Complexes?

**DOI:** 10.3390/molecules25122760

**Published:** 2020-06-15

**Authors:** Fabian Brunner, Alessandro Prescimone, Edwin C. Constable, Catherine E. Housecroft

**Affiliations:** Department of Chemistry, University of Basel, BPR 1096, Mattenstrasse 24a, CH-4058 Basel, Switzerland; fabian.brunner@unibas.ch (F.B.); alessandro.prescimone@unibas.ch (A.P.); edwin.constable@unibas.ch (E.C.C.)

**Keywords:** copper(I), bisphosphane, 2,2′-bipyridine, X-ray crystallography, photoluminescence, heteroleptic coordination compounds

## Abstract

The synthesis and structural characterization of 5,6′-dimethyl-2,2′-bipyridine (5,6′-Me_2_bpy) are reported, along with the preparations and characterizations of [Cu(POP)(5,6′-Me_2_bpy)][PF_6_] and [Cu(xantphos)(5,6′-Me_2_bpy)][PF_6_] (POP = bis(2-(diphenylphosphanyl)phenyl)ether, xantphos = 4,5-bis(diphenylphosphanyl)-9,9-dimethyl-9*H*-xanthene). Single-crystal X-ray structure determinations of [Cu(POP)(5,6′-Me_2_bpy)][PF_6_] and [Cu(xantphos)(5,6′-Me_2_bpy)][PF_6_] confirmed distorted tetrahedral copper(I) coordination environments with the 5-methylpyridine ring of 5,6′-Me_2_bpy directed towards the (C_6_H_4_)_2_O unit of POP or the xanthene unit of xantphos. In the xantphos case, this preference may be attributed to C–H…π interactions involving both the 6-CH unit and the 5-methyl substituent in the 5-methylpyridine ring and the arene rings of the xanthene unit. ^1^H NMR spectroscopic data indicate that this ligand orientation is also preferred in solution. In solution and the solid state, [Cu(POP)(5,6′-Me_2_bpy)][PF_6_] and [Cu(xantphos)(5,6′-Me_2_bpy)][PF_6_] are yellow emitters, and, for powdered samples, photoluminescence quantum yields (PLQYs) are 12 and 11%, respectively, and excited-state lifetimes are 5 and 6 μs, respectively. These values are lower than PLQY and τ values for [Cu(POP)(6,6′-Me_2_bpy)][PF_6_] and [Cu(xantphos)(6,6′-Me_2_bpy)][PF_6_], and the investigation points to the 6,6′-dimethyl substitution pattern in the bpy ligand being critical for enhancement of the PLQY.

## 1. Introduction

**Scheme 1:**
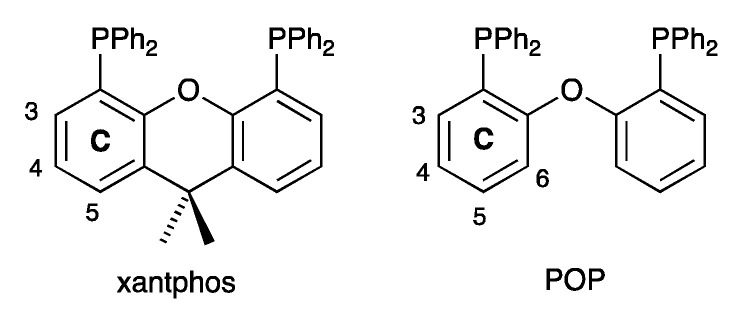
The structures of xantphos (4,5-bis(diphenylphosphanyl)-9,9-dimethyl-9*H*-xanthene, IUPAC PIN (9,9-dimethyl-9*H*-xanthene-4,5-diyl)bis(diphenylphosphane)) and POP (bis(2-(diphenylphosphanyl)phenyl)ether, IUPAC PIN oxydi(2,1-phenylene)]bis(diphenylphosphane)). The labelling scheme is used for the NMR spectroscopic assignments; the PPh_2_ phenyl rings are labelled **D**.

The development of solid-state lighting technologies has revolutionized modern domestic and commercial lighting, primarily through the development of devices which are cheaper to manufacture and operate [1]. Although light-emitting diodes (LEDs) and organic light-emitting diodes (OLEDs) lead the market, light-emitting electrochemical cells (LECs) offer a promising alternative [2,3]. LECs employ ionic transition metal compounds (iTMCs) as the light-emitting materials, the most commonly encountered of which are cyclometallated iridium(III) complexes [2,4] and heteroleptic [Cu(P^P)(N^N)]^+^ complexes [5] in which P^P is a wide bite-angle bisphosphane [6], such as xantphos and POP (Scheme 1), and N^N is typically a 2,2′-bipyridine (bpy) or 1,10-phenanthroline (phen) chelating ligand. This last class of compound follows from the seminal work of McMillin and coworkers, who observed that [Cu(P^P)(N^N)]^+^ complexes exhibit low-lying metal-to-ligand charge transfer (MLCT) excited states [7,8]. More recently, it has been demonstrated that many [Cu(P^P)(N^N)]^+^ complexes exhibit thermally activated delayed fluorescence (TADF) [9,10], and this has increased interest in this family of copper(I) emitters. Triplet and singlet excited states are statistically present in a 3:1 ratio, and, in TADF, there is a fast intersystem crossing from the lowest-lying singlet excited state (S_1_) to the triplet excited state (T_1_). The T_1_ state is long-lived, with a relatively slow phosphorescence. If the energy gap between the S_1_ and T_1_ states is small, thermal repopulation of the S_1_ state via a reverse intersystem crossing can occur, leading to enhanced fluorescence from the S_1_ state and an increased photoluminescence quantum yield (PLQY).

Since the potential for copper(I) iTMCs as light-emitting components in LECs was first demonstrated [11,12], xantphos and POP have been combined with many structurally and electronically diverse diimine ligands [5]. Some of the highest PLQY values and the best device electroluminescence (EL) performances have been achieved using bpy ligands containing simple substituents, including 6-alkyl and 6,6′-dialkyl groups [13,14], 6-alkyloxy and 6-alkylthio substituents [15], 6,6′-bis(alkyloxy) groups [16] and 6,6′-dihalo substituents [17]. Methyl substituents, in particular, lead to high PLQY values [13,14] and LEC performances. For example, a maximum luminance of 53 cd m^−2^ was observed in a LEC containing [Cu(POP)(6,6′-Me_2_bpy)][PF_6_] in the emissive layer [13]. Considering photoluminescence data for powdered samples, there is a significant improvement in quantum yield on going from [Cu(POP)(bpy)][PF_6_] (3% [17]), to [Cu(POP)(6-Mebpy)][PF_6_] (9.5% [13]) or [Cu(POP)(6,6′-Me_2_bpy)][PF_6_] (43.2% [13]), and from [Cu(xantphos)(bpy)][PF_6_] (1.7% [17]) to [Cu(xantphos)(6-Mebpy)][PF_6_] (34% [14]) or [Cu(xantphos)(6,6′-Me_2_bpy)][PF_6_] (37% [14]) (6-Mebpy = 6-methyl-2,2′-bipyridine, 6,6′-Me_2_bpy = 6,6′-dimethyl-2,2′-bipyridine). These data indicate that the introduction of at least one methyl group into the 6-position of bpy is beneficial for enhanced emission. In the case of the POP complex, there is a further enhancement when a second methyl group is introduced, but this is less pronounced in the case of the xantphos-containing analogue. Much of the effort in ligand design has emphasized electronic tuning through substituents attached at the 4- and 6-positions of the pyridine rings. In contrast, substitution at the 5-position has a similar inductive but a slightly different resonance electronic effect (6-Me, σ –0.17, σ_I_ –0.04, *σ*_R_ –0.04; 5-Me, σ –0.07, σ_I_ –0.04, *σ*_R_ –0.14) [18,19] but significantly changes the steric demands of the ligand. We were interested in comparing complexes containing the N^N ligand 5,6′-dimethyl-2,2′-bipyridine (5,6′-Me_2_bpy) with those containing 6-Mebpy or (isomeric) 6,6′-Me_2_bpy. Here we describe the synthesis, characterization, and photophysical and electrochemical properties of [Cu(POP)(5,6′-Me_2_bpy)][PF_6_] and [Cu(xantphos)(5,6′-Me_2_bpy)][PF_6_] and assess the effects of introducing the second methyl group into the 5- rather than the 6-position.

## 2. Results and Discussion

### 2.1. Synthesis and Structural Characterization of 5,6′-Me_2_bpy

**Scheme 2:**
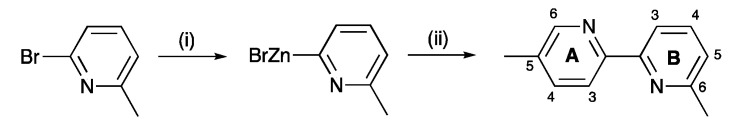
Synthesis of 5,6′-Me_2_bpy. Conditions: (**i**) *^n^*BuLi followed by ZnBr_2_, THF, −78 °C, 2 h; (**ii**) 2-bromo-5-methyl-2,2′-bipyridine, 5 mol% [Pd(PPh_3_)_4_], THF, 80 °C, 60 h. Ring labelling is for NMR spectroscopic assignments.

The ligand 5,6′-Me_2_bpy has previously been prepared in 66% overall yield using a Stille coupling [20,21] but we preferred to develop a tin-free approach. The palladium-catalyzed cross-coupling shown in Scheme 2 (in which the intermediate is made in situ and was not isolated) yielded 5,6′-Me_2_bpy, after work up, in 48% yield. Although the yield is lower than those reported for the Stille route [20,21], we consider that the procedure described here is advantageous in that no intermediate organometal reagent needs to be isolated. The ^1^H NMR spectrum (assigned using COSY and NOESY methods) agreed with that published [20], and the ^13^C{^1^H} NMR spectrum (not previously reported) was assigned using HMQC and HMBC methods.

**Figure 1:**
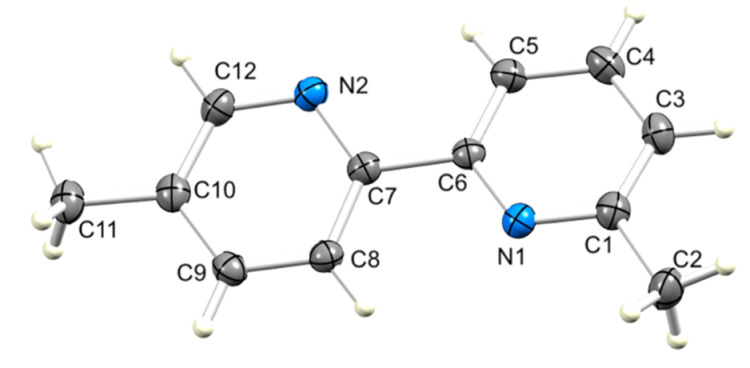
ORTEP representation of the structure of 5,6′-Me_2_bpy with ellipsoids plotted at a 40% probability level. Selected bond lengths: N1–C1 = 1.343(2), N1–C6 = 1.347(2), N2–C7 = 1.341(2), N2–C12 = 1.338(2), C1–C2 = 1.503(2), C10–C11 = 1.504(2) Å.

**Figure 2:**
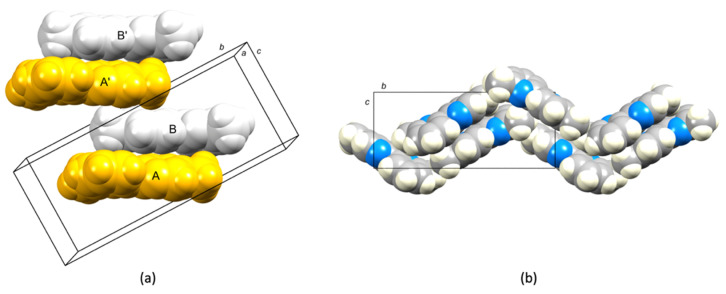
(**a**) Centrosymmetric pairs of 5,6′-Me_2_bpy molecules, colored by symmetry operation; see text for reference to the labels A, B, A′ and B′. (**b**) View down the *a*-axis showing zigzag chain arrangements of 5,6′-Me_2_bpy.

Single crystals of 5,6′-Me_2_bpy were obtained by diffusion of cyclohexane into an ethyl acetate solution of the compound. 5,6′-Me_2_bpy crystallizes in the monoclinic space group *P*2_1_/*n* and the structure is shown in Figure 1. The compound adopts the expected *trans*-conformation and the angle between the least-squares planes through the two pyridine rings is 16.5°. Bond lengths and angles are unexceptional, and selected bond lengths are listed in the caption to Figure 1. The molecules pack in centrosymmetric pairs (Figure 2a, pairs of molecules A/B, B/A’, and A’/B’). The first interaction involves face-to-face π-stacking of pyridine rings between molecules A/B and A’/B’. The distance between the planes of the pyridine rings containing N2 and N2i (symmetry code i = 1 – *x*, 1 – *y*, 1 – *z*) is 3.46 Å, and the intercentroid distance is 3.73 Å. For the second pairing (B/A’ in Figure 2a), a C_Me_–H…π interaction is important with a C_Me_…centroid distance of 3.51 Å. The shortest C_Me_…C_py_ distances are 3.68 and 3.69 Å, which are similar to the sum of the van der Waals radii for C_Me_ and C_sp2_ (3.70 Å) [22]. We consider the C_Me_…C_py_ rather than the H_Me_…C_py_ distances because the H atoms of the methyl group are in calculated positions. When the lattice is viewed along the *a*-axis, we observe an assembly into zigzag chains (Figure 2b). The latter features a close interaction between the 5-methyl substituent and the 6-substituted pyridine (py) ring of an adjacent molecule. The closest C_Me_…C_py_ separation is 3.79 Å, which is slightly longer than the sum of the C_Me_ and C_sp2_ van der Waals radii [22]. The relationship between the zigzag chains and the centrosymmetric pairs of molecules can be appreciated by comparing Figure 2a,b.

### 2.2. Synthesis and Characterization of [Cu(POP)(5,6′-Me_2_bpy)][PF_6_] and [Cu(xantphos)(5,6′-Me_2_bpy)][PF_6_]

The syntheses of [Cu(POP)(5,6′-Me_2_bpy)][PF_6_] and [Cu(xantphos)(5,6′-Me_2_bpy)][PF_6_] were carried out by either sequential (for POP) or simultaneous (for xantphos) addition of the bisphosphane and 5,6′-Me_2_bpy ligands to [Cu(MeCN)_4_][PF_6_]. This is a standard procedure for the different P^P ligands, and the reasons for the different strategies have previously been detailed [14,15]. The POP and xantphos-containing complexes were isolated in yields of 74.4% and 89.6%, respectively. The electrospray mass spectrum of each compound exhibited a base peak corresponding to the [M–PF_6_]^+^ ion (*m/z* 785.2 for [Cu(POP)(5,6′-Me_2_bpy)]^+^ and *m/z* 825.3 for [Cu(xantphos)(5,6′-Me_2_bpy)] (Figures S1 and S2; see the Supplementary Materials).

**Figure 3:**
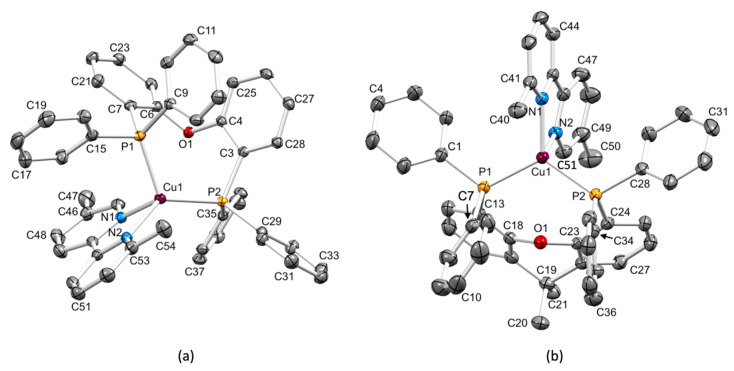
The structures of (**a**) the [Cu(POP)(5,6′-Me_2_bpy)]^+^ cation in [Cu(POP)(5,6′-Me_2_bpy)][PF_6_]·Me_2_CO and (**b**) the [Cu(xantphos)(5,6′-Me_2_bpy)]^+^ cation in [Cu(xantphos)(5,6′-Me_2_bpy)][PF_6_]·0.5CH_2_Cl_2_·0.75Et_2_O. Ellipsoids are plotted at a 40% probability level, and H atoms are omitted for clarity.

**Figure 4:**
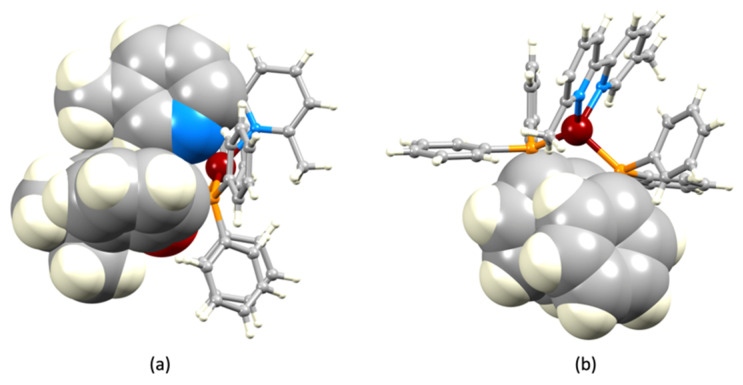
Space-filling representations are used to highlight (**a**) the accommodation of the C51–H51 unit (next to the 5-methyl substituent) within the bowl-shaped cavity of the xanthene unit in [Cu(xantphos)(5,6′-Me_2_bpy)]^+^, and (**b**) face-to-face π-stacking of the arene rings containing C3 and C9 in [Cu(POP)(5,6′-Me_2_bpy)]^+^.

Single crystals of [Cu(POP)(5,6′-Me_2_bpy)][PF_6_]·Me_2_CO and Cu(xantphos)(5,6′-Me_2_bpy)][PF_6_]·0.5CH_2_Cl_2_ 0.75Et_2_O were grown by diffusion of Et_2_O into an acetone or dichloromethane solution of the POP- and xantphos-containing compounds, respectively. The compounds crystallize in the monoclinic space groups *P*2_1_/*c* and *P*2_1_/*n,* respectively, and the structures of the cations are presented in Figure 3. Bond parameters for the copper(I) coordination spheres are listed in Table 1 along with Houser’s τ_4_ parameter [23]. The latter may be used to assess the distortion of the copper(I) centre away from *T*_d_ symmetry (τ_4_ = 1.00) towards *C*_3v_ (τ_4_ = 0.85), *C*_2v_ see-saw geometries (τ_4_ ≤ 0.64) and finally square-planar (τ_4_ = 0.00). The large P–Cu–N angles of 131.84(6)° in [Cu(POP)(5,6′-Me_2_bpy)]^+^ and 120.61(7)° and 116.05(7)° in [Cu(xantphos)(5,6′-Me_2_bpy)]^+^ contribute to values of τ_4_ (Table 1) that are close to τ_4_ = 0.85, calculated for a *C*_3v_ (trigonal pyramidal) geometry. In both complex cations, the 5,6′-Me_2_bpy ligand is positioned with the 5-methylpyridine ring facing the backbone of the bisphosphane ligand, and, in [Cu(xantphos)(5,6′-Me_2_bpy)]^+^, this results in the C51–H51 unit being accommodated within the bowl-shaped cavity of the xanthene unit (Figure 4a). Considering the centroids of the two outer rings of the xanthene unit, the C51–H51…centroid distances are 3.01 and 3.22 Å, consistent with C–H…π interactions [24]. We note that, in many of the crystal structures of [Cu(xantphos)(6-Xbpy)][PF_6_], in which 6-Xbpy is a 6-substituted-2,2′-bipyridine, the asymmetric 6-Xbpy ligand is oriented with the X group (X = Me, Et, OMe, OEt, OPh, SMe, SEt, SPh, Br [14,17,25,26]) lying over the xanthene bowl, rather than being remote from it. Examples of this alternative configuration are less commonly encountered in the solid state [14,27,28], and, in several structures, disordering indicates little energy difference between the two possible orientations [25,29]. Thus, it is noteworthy that the 5,6′-Me_2_bpy ligand exhibits a preference for an orientation with the 6-methyl group remote from the xanthene unit. A possible explanation is the combined effects of the C–H…π interactions described above and weak interactions between the 5-methyl substituent and the arene rings of the xanthene unit. The closest C_Me_…C_arene_ distance is 4.12 Å which, although greater than the sum of the C_Me_ and C_sp2_ van der Waals radii [22], may indicate a stabilizing influence. As in the earlier discussion of the structure of the free ligand, we choose to use the C_Me_…C_arene_ distance rather than the H_Me_…C_arene_ separation because the H atoms of the methyl group are in calculated positions. Intramolecular π-stacking interactions are considered to be important in [Cu(P^P)(N^N)]^+^ complexes in terms of contributing towards enhanced PLQY values [30], and, in [Cu(xantphos)(N^N)]^+^ cations, face-to-face π-stacking interactions are commonly observed between two phenyl rings of different PPh_2_ units [15]. However, in [Cu(xantphos)(5,6′-Me_2_bpy)]^+^, the angle between the planes of the phenyl rings containing atoms C7 and C34 (Figure 3b) is too large (39.5°) for a meaningful interaction. In contrast, in [Cu(POP)(N^N)]^+^ cations, π-stacking between a phenyl ring and one arene ring of the O(C_6_H_4_)_2_-unit in the POP ligand is often a feature in the solid state, and [Cu(POP)(5,6′-Me_2_bpy)]^+^ is no exception. Figure 4b shows the stacking of the rings containing atoms C3 and C9; the angle between the ring-planes is 14.7°, and the distance between the ring-centroids is 3.80 Å.

**Figure 5:**
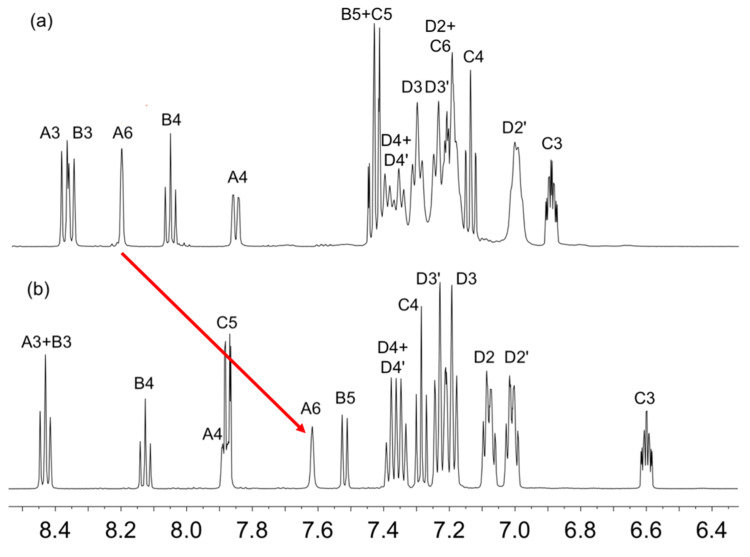
Comparison of the aromatic regions of the ^1^H NMR spectra (500 MHz, acetone-*d*_6_, 298 K) of (**a**) [Cu(POP)(5,6′-Me_2_bpy)][PF_6_] and (**b**) [Cu(xantphos)(5,6′-Me_2_bpy)][PF_6_]. See Scheme 1 and Scheme 2 for the atom labelling. Scale: *δ*/ppm.

**Figure 6:**
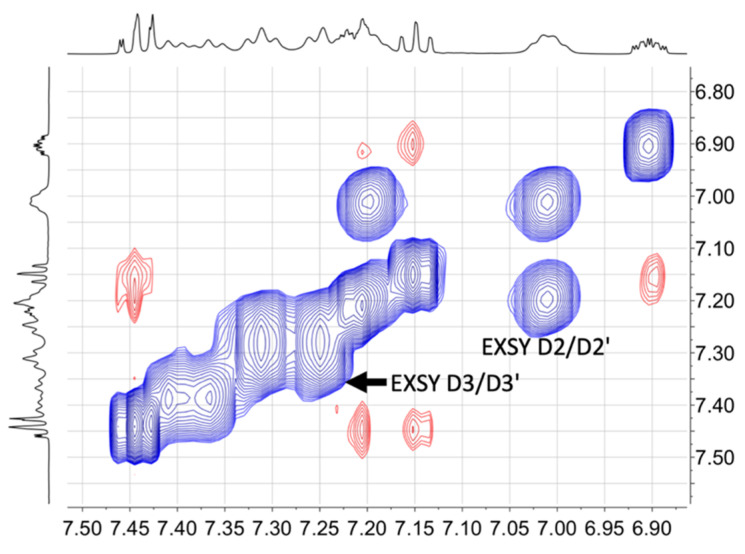
Part of the NOESY spectrum (500 MHz, acetone-*d*_6_, 298 K) of [Cu(POP)(5,6′-Me_2_bpy)][PF_6_] showing exchange (EXSY) peaks between pairs of phenyl rings D2/D2’ and D3/D3’.

The solid-state structures discussed above assist in an interpretation of the solution NMR spectroscopic properties of the copper(I) compounds. ^1^H, ^13^C{^1^H}, COSY, NOESY, HMBC and HMQC-NMR spectra were recorded, allowing full assignment of the ^1^H and ^13^C{^1^H} NMR resonances in the complexes. 1D and 2D spectra of the compounds are shown in Figure 5 and Figure 6, and in Figures S3–S10 in the Supplementary Materials. Figure 5 displays the aromatic regions of the ^1^H NMR spectra of the two compounds. Figure 5 reveals a substantial shift to lower frequency for the signal for proton A6 on going from [Cu(POP)(5,6′-Me_2_bpy)][PF_6_] to [Cu(xantphos)(5,6′-Me_2_bpy)][PF_6_]. This is consistent with the 5,6′-Me_2_bpy ligand adopting the same configuration in solution as in the solid state, such that proton A6 is affected by the ring currents of the two arene rings of the xanthene unit. The appearance of two sets of signals for the PPh_2_ phenyl D-rings (labelled D and D’ in Figure 5 and Figure S5) is consistent with ^1^H and ^13^C-NMR spectroscopic data for related POP and xantphos-containing copper(I) complexes; see, for example, [14,15,28,31,32]. In [Cu(POP)(5,6′-Me_2_bpy)][PF_6_], exchange (EXSY) peaks are observed between pairs of protons D2/D2’ and D3/D3’ (Figure 6). Corresponding peaks are not observed in the xantphos-containing complex (Figure S10). NOESY crosspeaks are observed in [Cu(POP)(5,6′-Me_2_bpy)][PF_6_] between protons D2/A6 and D2’/A6, and between D2/Me-B6 and D2’/Me-B6, indicating that the flexible POP backbone undergoes dynamic behavior on the NMR timescale at 298 K, which exchanges the axial and equatorial phenyl rings of each PPh_2_ unit [14,28].

### 2.3. Electrochemical and Photophysical Properties

**Figure 7:**
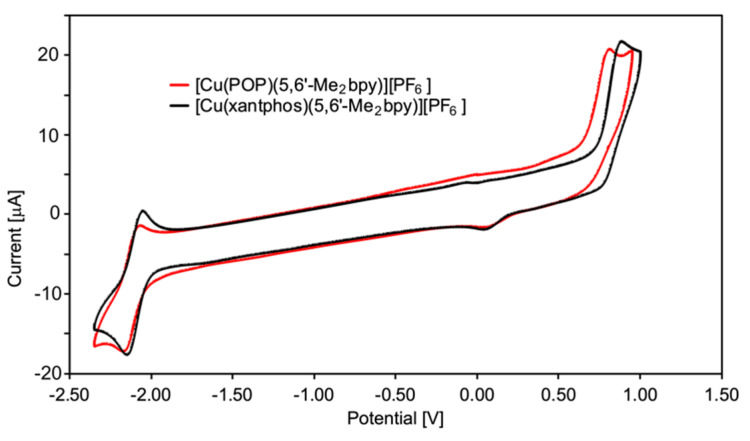
Cyclic voltammograms of [Cu(POP)(5,6′-Me_2_bpy)][PF_6_] and [Cu(xantphos)(5,6′-Me_2_bpy)][PF_6_] in propylene carbonate at a scan rate of 0.1 V s^−1^ referenced to internal Fc/Fc^+^ = 0 V. The second of three reproducible cycles is shown.

Both [Cu(POP)(5,6′-Me_2_bpy)][PF_6_] and [Cu(xantphos)(5,6′-Me_2_bpy)][PF_6_] are redox active and their electrochemical behavior was investigated in propylene carbonate solution using cyclic voltammetry (Figure 7). Each of [Cu(POP)(5,6′-Me_2_bpy)][PF_6_] and [Cu(xantphos)(5,6′-Me_2_bpy)][PF_6_] exhibits an irreversible copper-centered oxidation with *E*_pc_ values of +0.81 and 0.89 V, respectively, and a reversible bpy-centered reductive process with *E*_1/2_ values of −2.12 V and −2.10 V, respectively (*E*_pa_ − *E*_pc_ = 100 V in each case). Data for the oxidative process are presented in Table 2 and are compared with oxidation potentials for closely related compounds. Note that a common solvent was not used for all the compounds in Table 2. Nonetheless, the data for the xantphos-containing complexes are consistent with a trend of copper(I) oxidation occurring at the highest potential for [Cu(xantphos)(6,6′-Me_2_bpy)]^+^ and following a sequence according to the N^N ligand of 6,6′-Me_2_bpy > 5,6′-Me_2_bpy > 6-Mebpy > bpy. Copper(I)-to-copper(II) oxidation is accompanied by a geometrical change from tetrahedral to square-planar, and the trend in the oxidation potentials is consistent with decreasing steric hindrance in the coordination sphere of the copper center along the series from 6,6′-Me_2_bpy to 5,6′-Me_2_bpy to 6-Mebpy to bpy. The 5,6′-Me_2_bpy also fits into this pattern for the POP-containing compounds, although (from the literature data) the trend from 6-Mebpy to bpy is less well defined than for the xantphos family of compounds (Table 2).

**Figure 8:**
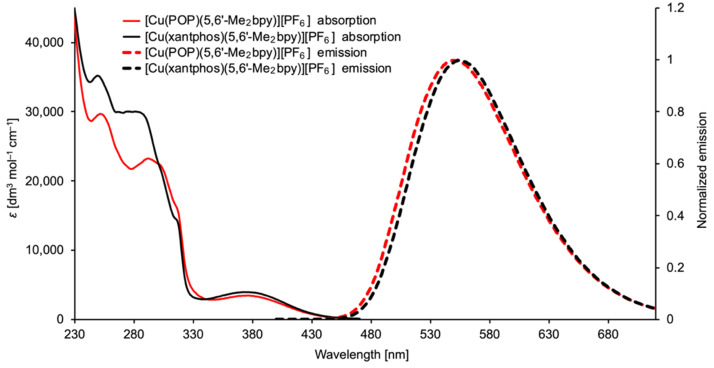
Solution absorption spectra of [Cu(POP)(5,6′-Me_2_bpy)][PF_6_] and [Cu(xantphos)(5,6′-Me_2_bpy)][PF_6_] in CH_2_Cl_2_ (2.5 × 10^–5^ mol dm^–3^) (solid lines) and solid-state emission spectra (dotted lines).

The solution absorption spectra of the heteroleptic copper(I) compounds (Figure 8) show intense absorptions below 330 nm arising from ligand-based π*←π transitions. The broad absorption band with *λ*_max_ at 376 nm in [Cu(POP)(5,6′-Me_2_bpy)][PF_6_] and at 374 nm in [Cu(xantphos)(5,6′-Me_2_bpy)][PF_6_] arises from MLCT transitions. The MLCT *λ*_max_ values for the 5,6′-Me_2_bpy complexes are similar to those for [PF_6_]^−^ or [BF_4_]^–^ salts of [Cu(POP)(6,6′-Me_2_bpy)]^+^ (372 nm [33]) and [Cu(xantphos)(6,6′-Me_2_bpy)]^+^ (375 nm [33], 378 nm [14]), but are blue-shifted with respect to those of salts of [Cu(POP)(bpy)]^+^ (389 nm [12], 385 nm [33]), [Cu(xantphos)(bpy)]^+^ (383 nm [29], 382 nm [33]), [Cu(POP)(6-Mebpy)]^+^ (380 nm [13]) and [Cu(xantphos)(6-Mebpy)]^+^ (379 nm [14]). Since the LUMO of a [Cu(P^P)(N^N)]^+^ complex is localized on the N^N ligand [14], the general trends in *λ*_max_ are consistent with a destabilization of the LUMO upon introducing electron-donating methyl substituents into the bpy domain.

Both aerated and deaerated solutions of [Cu(POP)(5,6′-Me_2_bpy)][PF_6_] and [Cu(xantphos)(5,6′-Me_2_bpy)][PF_6_] are weakly emissive when excited into the MLCT band at 375 and 374 nm, respectively. Values of *λ*_em_^max^ were 626 and 622 nm (yellow emitters), respectively, PLQY values were around 1%, and excited state lifetimes (τ) ≤ 1 μs. Low PLQYs in solution, even in non-coordinating solvents, have been attributed to the copper(I) complex undergoing exciplex formation, which favors non-radiative decay [35]. Excitation of powdered samples of the compounds (λexc = 365 nm) leads to emission maxima at higher energies than the solution samples while maintaining a yellow emission color. Such a blue-shift is typical of [Cu(P^P)(N^N)]^+^ complexes [36,37]. Table 3 summarizes the solid-state emission data, and the emission spectra are shown in Figure 8. Values of PLQY and τ for [Cu(POP)(5,6′-Me_2_bpy)][PF_6_] and [Cu(xantphos)(5,6′-Me_2_bpy)][PF_6_] are lower than for their 6,6′-Me_2_bpy-containing analogues (Table 3). The blue-shift in the emission maximum on going from the heteroleptic complexes containing bpy to those with 6-Mebpy, 5,6′-Me_2_bpy or 6,6′-Me_2_bpy is consistent with the electron-donating character of the methyl substituents. However, comparing values of λ^em^_max_ for [Cu(P^P)(5,6′-Me_2_bpy)][PF_6_] and [Cu(P^P)(6,6′-Me_2_bpy)][PF_6_] indicates that a red-shift in the emission accompanies a change from 6,6′-Me_2_bpy to 5,6′-Me_2_bpy, and this in turn is consistent with the introduction of an electron-donating methyl group in a position *meta* to the N-donor stabilizing the LUMO of the complex. The PLQY values in Table 3 underline the importance of a second methyl group adjacent to the coordination site of the bpy ligand.

## 3. Materials and Methods

### 3.1. General

^1^H, ^13^C{^1^H} and ^31^P{^1^H} NMR spectra were recorded at 298 K on a Bruker Avance III-500 NMR spectrometer (Bruker BioSpin AG, Fällanden, Switzerland). ^1^H and ^13^C-NMR chemical shifts were referenced to residual solvent peaks with respect to *δ*(TMS) = 0 ppm and ^31^P NMR chemical shifts with respect to *δ*(85% aqueous H_3_PO_4_) = 0 ppm. A Shimadzu LCMS-2020 (Shimadzu Schweiz GmbH, 4153 Reinach, Switzerland) was used to record electrospray ionization (ESI) mass spectra. Solution absorption and emission spectra were recorded using Shimadzu UV-2600 and Shimadzu RF-6000 instruments (Shimadzu Schweiz GmbH, 4153 Reinach, Switzerland), respectively. A Hamamatsu absolute photoluminescence quantum yield spectrometer C11347 Quantaurus-QY was used to measure PLQYs, and emission lifetimes and powder emission spectra were measured using a Hamamatsu Compact Fluorescence Lifetime Spectrometer C11367 Quantaurus-Tau with an LED light source (*λ*_exc_ = 365 nm).

Electrochemical measurements were carried out using a CH Instruments 900B potentiostat (CH Instruments, Texas 78738, USA) with [*^n^*Bu_4_N][PF_6_] (0.1 M) as supporting electrolyte and at a scan rate of 0.1 V s^–1^. The working electrode was glassy carbon, the reference electrode was a leakless Ag^+^/AgCl (eDAQ ET069-1) (eDAQ Europe, 01-471 Warszawa, Poland), and the counter-electrode was a platinum wire. Final potentials were internally referenced with respect to the Fc/Fc^+^ couple.

POP and xantphos were purchased from Acros (Fisher Scientific AG, 4153 Reinach Switzerland) and Fluorochem (Chemie Brunschwig AG, 4052 Basel, Switzerland), respectively. [Cu(MeCN)_4_][PF_6_] was prepared by the literature route [38].

### 3.2. Synthesis of 5,6′-Me_2_bpy

*n*BuLi (4.66 mL, 11.7 mmol, 2.5 M in *n*-hexane, 1.1 eq.) was added to a degassed solution of 2-bromo-6-methylpyridine (2.01 g, 11.7 mmol, 1.1 eq.) in dry THF (10 mL) at −78 °C under a nitrogen atmosphere. The mixture was stirred for 30 min during which time it turned deep red. Then, a solution of ZnCl_2_ in Et_2_O (10.6 mL, 10.6 mmol, 1.0 M in Et_2_O, 1.0 eq.) was added dropwise and the reaction mixture was stirred for 1.5 h. It was then allowed to warm to ambient temperature (ca. 22 °C) and was added dropwise to a solution of 2-bromo-5-methylpyridine (20.1 g, 11.7 mmol, 1.1 eq.) in dry THF (30 mL) containing [Pd(PPh_3_)_4_] (612 mg, 0.53 mmol, 0.05 eq.). This reaction mixture was heated to 80 °C and this temperature was maintained with stirring for 60 h. After cooling to ambient temperature, an aqueous solution of NaOH (ca. 100 mL, 3 M) was added until most of the precipitate had dissolved. The mixture was extracted with CH_2_Cl_2_ (3 × 50 mL) and the combined organic fractions were washed with aqueous NaOH, and then dried over MgSO_4_. The solvent was then removed under reduced pressure. The crude material was purified by column chromatography (neutral alumina, cyclohexane:ethyl acetate 40:1, followed by a second column with silica, cyclohexane:ethyl acetate 20:1) to give 5,6′-Me_2_bpy (937 mg, 5.08 mmol, 48%) as a white solid. ^1^H NMR (500 MHz, CDCl_3_) *δ*/ppm: 8.52–8.48 (m, 1H, H^A6^), 8.29 (d, *J* = 8.1 Hz, 1H, H^A3^), 8.13 (d, *J* = 7.9 Hz, 1H, H^B3^), 7.68 (t, *J* = 7.7 Hz, 1H, H^B4^), 7.61 (ddd, *J* = 8.1, 2.2, 0.7 Hz, 1H, H^A4^), 7.14 (d, *J* = 7.6 Hz, 1H, H^B5^), 2.63 (s, 3H, H^Me-B6^), 2.39 (s, 3H, H^Me-A5^). ^13^C{^1^H} NMR (126 MHz, CDCl_3_) *δ*/ppm: 158.0 (C^B6^), 155.8 (C^B2^), 154.0 (C^A2^), 149.7 (C^A6^), 137.6 (C^A4^), 137.2 (C^B4^), 133.3 (C^A5^), 123.1 (C^B5^), 120.9 (C^A3^), 118.0 (C^B3^), 24.8 (C^MeB6^), 18.5 (C^MeA5^). ESI MS: *m/z* 184.1 [M + H]^+^ (base peak, calc. 184.1). Found: C 78.01, H 6.75, N 15.40; C_12_H_12_N_2_ requires C 78.23, H 6.57, N 15.21%.

### 3.3. [Cu(POP)(5,6′-Me_2_bpy)][PF_6_]

[Cu(MeCN)_4_][PF_6_] (93.2 mg, 0.250 mmol, 1.0 eq.) and POP (148 mg, 0.275 mmol, 1.1 eq.) were dissolved in CH_2_Cl_2_ (30 mL) and the reaction mixture was stirred for 1.5 h at room temperature (ca. 22 °C). 5,6′-Me_2_bpy (46.1 mg, 0.250 mmol, 1.0 eq.) was then added, and stirring was continued for another 1.5 h. The yellow solution was filtered, and the solvent volume of the filtrate was reduced (under reduced pressure) and added to *n-*hexane (ca. 40 mL) to precipitate the product. The precipitate was separated and was washed with *n-*hexane (4 × 10 mL) using sonication and dried under vacuum. [Cu(POP)(5,6′-Me_2_bpy)][PF_6_] was isolated as a yellow solid (173 mg, 0.186 mmol, 74.4%). ^1^H NMR (500 MHz, acetone-*d*_6_) *δ*/ppm: 8.39 (d, *J* = 8.4 Hz, 1H, H^A3^), 8.36 (d, *J* = 8.3 Hz, 1H, H^B3^), 8.21 (s, 1H, H^A6^), 8.06 (t, *J* = 7.8 Hz, 1H, H^B4^), 7.86 (dd, *J* = 8.4, 2.1 Hz, 1H, H^A4^), 7.48–7.42 (m, 3H, H^B5+C5^), 7.42–7.33 (m, 4H, H^D4+D4’^), 7.31 (t, *J* = 7.5 Hz, 4H, H^D3^), 7.28–7.18 (m, 10H, H^C6+D2+D3’^), 7.15 (td, *J* = 7.5, 1.1 Hz, 2H, H^C4^), 7.05–6.97 (m, 4H, H^D2’^), 6.90 (m, 2H, H^C3^), 2.48 (s, 3H, H^Me-B6^), 2.11 (s, 3H, H^Me-A5^). ^13^C{^1^H} NMR (126 MHz, acetone-*d*_6_) *δ*/ppm: 159.6 (C^B6^), 158.9 (t, *J*_PC_ = 6 Hz, C^C1^), 152.8 (t, *J*_PC_ = 2 Hz, C^B2^), 150.9 (t, *J*_PC_ = 2 Hz, C^A2^), 150.4 (C^A6^), 140.0 (C^B4^), 139.9 (C^A4^), 137.0 (C^A5^) 135.0 (C^C3^), 134.2 (t, *J*_PC_ = 8 Hz, C^D2^), 133.7 (t, *J*_PC_ = 8 Hz, C^D2’^), 133.2 (C^C5^), 132.1 (overlapping C^D1’+A3D1^), 131.2 (C^D4^), 130.8 (C^D4’^), 129.8 (t, *J*_PC_ = 4 Hz, C^D3^), 129.6 (t, *J*_PC_ = 4 Hz, C^D3’^), 126.6 (C^B5^), 126.1 (t, *J*_PC_ = 2 Hz, C^C4^), 125.0 (overlapping d, *J*_PC_ = 14 Hz, C^C2^), 123.0 (C^A3^), 121.2 (C^C6^), 120.5 (C^B3^), 26.8 (C^Me-B6^), 18.2 (C^Me-A5^). ^31^P{^1^H} NMR (202 MHz, acetone-*d*_6_) *δ*/ppm: –12.5 (broad, FWHM ≈ 450 Hz), –144.2 (septet, *J*_PF_ = 707 Hz, [PF_6_]^–^). ESI-MS: *m/z* 785.20 [M–PF_6_]^+^ (base peak, calc. 785.19), 601.05 [Cu(POP)]^+^ (calc. 601.09). UV-Vis (CH_2_Cl_2_, 2.5 × 10^–5^ mol dm^–3^): *λ*/nm (*ε*/dm^3^ mol^–1^ cm^–1^) 253 (29,600), 293 (23,000), 316 (16,000), 376 (3,400). Found: C 62.03, H 4.87, N 2.68; C_48_H_40_CuF_6_N_2_OP_3_ requires C 61.90, H 4.33, N 3.01%.

### 3.4. [Cu(xantphos)(5,6′-Me_2_bpy)][PF_6_]

[Cu(MeCN)_4_][PF_6_] (93.2 mg, 0.250 mmol, 1.0 eq.) was dissolved in CH_2_Cl_2_ (15 mL). A solution of xantphos (145 mg, 0.250 mmol, 1.0 eq.) and 5,6′-Me_2_bpy (46.1 mg, 0.250 mmol, 1.0 eq.) was added and the mixture turned orange then yellow while it was stirred for 1.5 h at room temperature (ca. 22 °C). The yellow solution was filtered, and the solvent volume was reduced under vacuum. Et_2_O was then added to precipitate the product, and the solid was collected by filtration, washed with Et_2_O (4 × 10 mL) using sonication, and dried under vacuum. [Cu(5,6′-Me_2_bpy)(xantphos)][PF_6_] was isolated as a yellow solid (218 mg, 0.224 mmol, 89.6%). ^1^H NMR (500 MHz, acetone-*d*_6_) *δ*/ppm: 8.45 (d, *J* = 8.0 Hz, 1H, H^A3^), 8.43 (d, *J* = 7.5 Hz, 1H, H^B3^), 8.13 (t, *J* = 7.8 Hz, 1H, H^B4^), 7.90−7.86 (overlapping m, 3H, H^A4+C5^), 7.62 (s, 1H, H^A6^), 7.52 (d, *J* = 7.7 Hz, 1H, H^B5^), 7.38 (t, *J* = 7.6 Hz, 2H, H^D4/D4’^), 7.35 (t, *J* = 7.2 Hz, 2H, H^D4/D4’^), 7.29 (t, *J* = 7.7 Hz, 2H, H^C4^), 7.23 (m, 4H, H^D3’^), 7.20 (m, 4H, H^D3^), 7.08 (m, 4H, H^D2^), 7.01 (m, 4H, H^D2’^), 6.60 (dtd, *J* = 7.6, 3.8, 1.4 Hz, 2H, H^C3^), 2.40 (s, 3H, H^Me-B6^), 2.05 (s, 3H, H^Me-A5^), 1.87 (s, 3H, H^Me-xantphos^), 1.77 (s, 3H, H^Me-xantphos^). ^13^C{^1^H} NMR (126 MHz, acetone-*d*_6_) *δ*/ppm: 159.2 (C^B6^), 155.9 (t, *J*_PC_ = 6 Hz, C^C1^), 152.5 (t, *J*_PC_ = 2 Hz, C^B2^), 151.0 (t, *J*_PC_ = 2 Hz, C^A2^), 149.6 (C^A6^), 140.3 (C^B4^), 140.2 (C^A4^), 137.1 (C^A5^), 135.1 (t, *J*_PC_ = 2 Hz, C^C6^), 133.8 (t, *J*_PC_ = 8 Hz, C^D2’^), 133.6 (t, *J*_PC_ = 8 Hz, C^D2^), 132.6 (t, *J*_PC_ = 17 Hz, C^D1’^), 132.3 (t, *J*_PC_ = 17 Hz, C^D1^), 131.6 (C^C3^), 131.2 (C^D4’^), 131.0 (C^D4^), 129.8 (t, *J*_PC_ = 5 Hz, C^D3’^), 129.8 (t, *J*_PC_ = 5 Hz, C^D3^), 128.4 (C^C5^), 126.6 (C^B5^), 126.4 (t, *J*_PC_ = 2.4 Hz, C^C4^), 123.3 (C^A3^), 121.4 (overlapping d, *J*_PC_ = 14 Hz, C^C2^), 120.7 (C^B3^), 37.0 (C^Cq-xantphos^), 28.7 (C^Me-xantphos^), 27.8 (C^Me-xantphos^), 26.9 (C^Me-B6^), 18.1 (C^Me-A5^). ^31^P{^1^H} NMR (202 MHz, acetone-*d*_6_) *δ*/ppm: −12.1 (broad, FWHM ≈ 330 Hz), −144.2 (septet, *J*_PF_ = 707 Hz, [PF_6_]^−^). ESI-MS: *m/z* 825.25 [M − PF_6_]^+^ (base peak, calc. 825.22), 641.10 [Cu(xantphos)]^+^ (calc. 641.12). UV-Vis (CH_2_Cl_2_, 2.5 × 10^–5^ mol dm^–3^): *λ*/nm (*ε*/dm^3^ mol^–1^ cm^–1^) 251 (35,000), 287 (29,700), 315 (14,000), 374 (3900). Found: C 62.82, H 4.54, N 2.97; C_51_H_44_CuF_6_N_2_OP_3_ requires C 63.06, H 4.57, N 2.88%

### 3.5. Crystallography

Single-crystal data for the ligand were collected on a STOE StadiVari diffractometer equipped with a Pilatus300K detector and a Metaljet D2 source (GaKα radiation). Data reduction used STOE software [39] and the structure was solved using Olex2 [40], ShelXT [41], and ShelXL v. 2014/7 [42]. For the copper(I) compounds, single-crystal data were collected on a Bruker APEX-II diffractometer (CuKα radiation) with data reduction, solution and refinement using the programs APEX2 [43], Superflip [44,45] and CRYSTALS [46]. Structure analysis including the ORTEP diagrams employed the program Mercury CSD v. 4.1.1 [47,48]. For [Cu(xantphos)(5,6′-Me_2_bpy)][PF_6_], SQUEEZE [49] was used to treat part of the solvent region, and an additional 0.75 Et_2_O was found that sums with the 0.5 CH_2_Cl_2_ that could be refined.

### 3.6. 5,6′-Me_2_bpy

C_12_H_12_N_2_, *M*_r_ = 184.24, colorless plate, monoclinic, space group *P*2_1_/*n*, *a* = 6.4300(2), *b* = 19.2147(4), *c* = 8.0931(2) Å, β = 91.147(2)^o^, *V* = 999.71(4) Å^3^, *D*_c_ = 1.224 g cm^–3^, *T* = 130 K, *Z* = 4, μ(GaKα) = 0.369 mm^−1^. Total 36,616 reflections, 2030 unique (*R*_int_ = 0.0291). Refinement of 1952 reflections (130 parameters) with *I* > 2σ(*I*) converged at final *R*_1_ = 0.0564 (*R*_1_ all data = 0.0574), *wR*_2_ = 0.1686 (*wR*_2_ all data = 0.1698), gof = 1.190. CCDC 2005673.

### 3.7. [Cu(POP)(5,6′-Me_2_bpy)][PF_6_]^.^Me_2_CO

C_51_H_46_CuF_6_N_2_O_2_P_3_, *M*_r_ = 989.39, orange needle, monoclinic, space group *P*2_1_/*c*, *a* = 9.5613(9), *b* = 14.8515(13), *c* = 32.472(3) Å, β = 90.338(4)^o^, *V* = 4611.0(7) Å^3^, *D*_c_ = 1.425 g cm^–3^, *T* = 130 K, *Z* = 4, μ(CuKα) = 2.227 mm^–1^. Total 31,246 reflections, 8211 unique (*R*_int_ = 0.029). Refinement of 7611 reflections (586 parameters) with *I* > 2σ(*I*) converged at final *R*_1_ = 0.0392 (*R*_1_ all data = 0.0420), *wR*_2_ = 0.0529 (*wR*_2_ all data = 0.0536), gof = 0.9967. CCDC 2005674.

### 3.8. [Cu(xantphos)(5,6′-Me_2_bpy)][PF_6_] ^.^0.5CH_2_Cl_2_^.^0.75Et_2_O

C_54.50_H_52.50_ClCuF_6_N_2_O_1.75_P_3_, *M*_r_ = 1069.38, yellow block, monoclinic, space group *P*2_1_/*n*, *a* = 17.6502(15), *b* = 16.8504(15), *c* = 18.4582(16) Å, β = 104.685(3)^o^, *V* = 5310.4(8) Å^3^, *D*_c_ = 1.338 g cm^–3^, *T* = 130 K, *Z* = 4, μ(CuKα) = 2.422 mm^–1^. Total 55,278 reflections, 9603 unique (*R*_int_ = 0.0319). Refinement of 8839 reflections (608 parameters) with *I* > 2σ(*I*) converged at final *R*_1_ = 0.0544 (*R*_1_ all data = 0.0575), *wR*_2_ = 0.1728 (*wR*_2_ all data = 0.1768), gof = 1.025. CCDC 2005675.

## 4. Conclusions

We have reported the synthesis and structural characterization of the 5,6′-Me_2_bpy ligand, and the preparations and solution and solid-state characterizations of the heteroleptic [Cu(POP)(5,6′-Me_2_bpy)][PF_6_] and [Cu(xantphos)(5,6′-Me_2_bpy)][PF_6_] compounds. Crystallographic structure determinations of the complexes confirmed distorted tetrahedral copper(I) coordination environments with the 5,6′-Me_2_bpy ligand oriented with the 5-methylpyridine ring directed towards the (C_6_H_4_)_2_O unit of POP or the xanthene unit of xantphos. In the latter, this preference appears to arise from C–H…π interactions involving both the 6-CH unit and the 5-methyl substituent in the 5-methylpyridine ring and the arene rings of the xanthene unit. ^1^H NMR spectroscopic data are consistent with this same orientation being preferred in solution. The electrochemical behavior of [Cu(POP)(5,6′-Me_2_bpy)][PF_6_] and [Cu(xantphos)(5,6′-Me_2_bpy)][PF_6_] was investigated and the copper(I) oxidation occurs at a lower potential than in corresponding complexes with 6,6′-Me_2_bpy. [Cu(POP)(5,6′-Me_2_bpy)][PF_6_] and [Cu(xantphos)(5,6′-Me_2_bpy)][PF_6_] are yellow emitters in both solution and the solid-state, and, for powdered samples, PLQY values of 12 and 11%, respectively, and excited-state lifetimes of 5 and 6 μs, respectively, were observed. These values are lower than the PLQY and τ values for [Cu(POP)(6,6′-Me_2_bpy)][PF_6_] and [Cu(xantphos)(6,6′-Me_2_bpy)][PF_6_] [13,14]. Our results underline the importance of the 6,6′-dimethyl substitution pattern in the bpy ligand for enhancement of the PLQY in particular.

## Figures and Tables

**Table 1:** Bond lengths and angles in the coordination sphere of each [Cu(P^P)(5,6′-Me_2_bpy)]^+^ cation, and values of the τ_4_ parameter.

Parameter	[Cu(POP)(5,6′-Me_2_bpy)]^+^	[Cu(xantphos)(5,6′-Me_2_bpy)]^+^
Cu–N/Å	2.075(2), 2.089(2)	2.053(2), 2.105(2)
Cu–P/Å	2.2967(7), 2.2517(7)	2.2488(8), 2.2555(8)
P–Cu–P/^o^	113.48(3)	117.36(3)
N–Cu–N/^o^	79.90(8)	80.02(9)
N–Cu–P/^o^	131.84(6), 108.61(6), 110.64(6), 103.82(6)	120.61(7), 116.05(7), 103.55(7), 110.47(7)
τ_4_^1^	0.83	0.87

^1^ τ_4_ parameter, see reference [23].

**Table 2:** Copper(I)/(II) oxidation potentials in [Cu(POP)(5,6′-Me_2_bpy)][PF_6_] and [Cu(xantphos)(5,6′-Me_2_bpy)][PF_6_] in propylene carbonate solutions (ca. 10^−4^ mol dm^–3^); values are referenced to internal Fc/Fc^+^ = 0.0 V; [*^n^*Bu_4_N][PF_6_] as supporting electrolyte and a scan rate of 0.1 V s^–1^. Values are compared to literature data (versus Fc/Fc^+^) for related compounds; see footnotes for solvents.

Compound	*E*_1/2_ /V	*E*_pc_–*E*_pa_/mV	*E*_pc_^a^/V	Reference
[Cu(POP)(5,6′-Me_2_bpy)][PF_6_]			+0.81	This work
[Cu(xantphos)(5,6′-Me_2_bpy)][PF_6_]			+0.89	This work
[Cu(POP)(6,6′-Me_2_bpy)][BF_4_]	+0.82 ^b^	– ^d^		[33]
[Cu(xantphos)(6,6′-Me_2_bpy)][PF_6_]	+0.90	150		[29]
[Cu(POP)(6-Mebpy)][PF_6_]	+0.69	– ^d^		[13]
[Cu(xantphos)(6-Mebpy)][PF_6_]	+0.85 ^c^	100		[34]
[Cu(POP)(bpy)][PF_6_]	+0.72	110		[29]
[Cu(POP)(bpy)][BF_4_]	+0.72 ^b^	– ^d^		[33]
[Cu(xantphos)(bpy)][PF_6_]	+0.76	110		[29]
[Cu(xantphos)(bpy)][BF_4_]	+0.67 ^b^	– ^d^		[33]

^a^ The value of *E*_pc_ is stated when the process is irreversible. ^b^ Recorded in MeCN. ^c^ Recorded in CH_2_Cl_2_. ^d^ No *E*_pc_ − *E*_pa_ value reported.

**Table 3:** Solid-state emission data (298 K) for the copper(I) compounds (λexc = 365 nm) compared to data for related compounds.

Compound	λ^em^_max_/nm	PLQY/% ^a^	τ/μs ^a^	Reference
[Cu(POP)(5,6′-Me_2_bpy)][PF_6_]	553	12	6 ^b^	This work
[Cu(xantphos)(5,6′-Me_2_bpy)][PF_6_]	555	11	5 ^b^	This work
[Cu(POP)(6,6′-Me_2_bpy)][PF_6_]	535	43.2	10.5 ^c^	[13]
[Cu(xantphos)(6,6′-Me_2_bpy)][PF_6_]	539	37	11 ^c^	[14]
[Cu(POP)(6-Mebpy)][PF_6_]	567	9.5	2.6 ^b^	[13]
[Cu(xantphos)(6-Mebpy)][PF_6_]	547	34	9.6 ^b^	[14]
[Cu(POP)(bpy)][PF_6_]	580	3.0	1.5 ^c^	[17]
[Cu(xantphos)(bpy)][PF_6_]	587	1.7	1.5 ^c^	[17]

^a^Values from the literature are quoted as reported, hence the inconsistency in the number of significant figures. ^b^A biexponential fit to the decay was used, using the equation: *τ* = Σ*A_i_τ_i_*/Σ*A_i_* where *A_i_* is the pre-exponential factor for the lifetime. Values of *A*_1_, *A*_2_, τ_1_ and τ_2_ are given in Table S1. ^c^A monoexponential fit to the decay was used.

## Supplementary Materials

The following are available online. Figures S1 and S2: mass spectra. Figures S3–S10: NMR spectra. Table S1: Parameters for biexponential fit to the lifetime decays.
