# Supplementary material for: Positional Isomerism in the N^N Ligand: How Much Difference Does a Methyl Group Make in [Cu(P^P)(N^N)]+ Complexes?
Source: Molecules. 2020 Jun 15;25(12):2760. doi: 10.3390/molecules25122760 (PMC7356218; doi:10.3390/molecules25122760)
Supplement: Supplementary file 1 [file molecules-25-02760-s001.zip › Supporting Information.docx]

Supporting Material to accompany:

Positional isomerism in the N^N ligand: How much difference does a methyl group make in [Cu(P^P)(N^N)]^+^ complexes?

Fabian Brunner ^1^, Alessandro Prescimone ^1^, Edwin C. Constable ^1^ , Catherine E. Housecroft ^1^*

^1^Department of Chemistry, University of Basel, BPR 1096, Mattenstrasse 24a, CH-4058 Basel, Switzerland; fabian.brunner@unibas.ch (F.B.); alessandro.prescimone@unibas.ch (A.P.); edwin.constable@unibas.ch (E.C.C.)

Fig. S1. ESI-MS of [Cu(POP)(5,6'-Me_2_bpy)][PF_6_].

Fig. S2. ESI-MS of [Cu(xantphos)(5,6'-Me_2_bpy)][PF_6_].


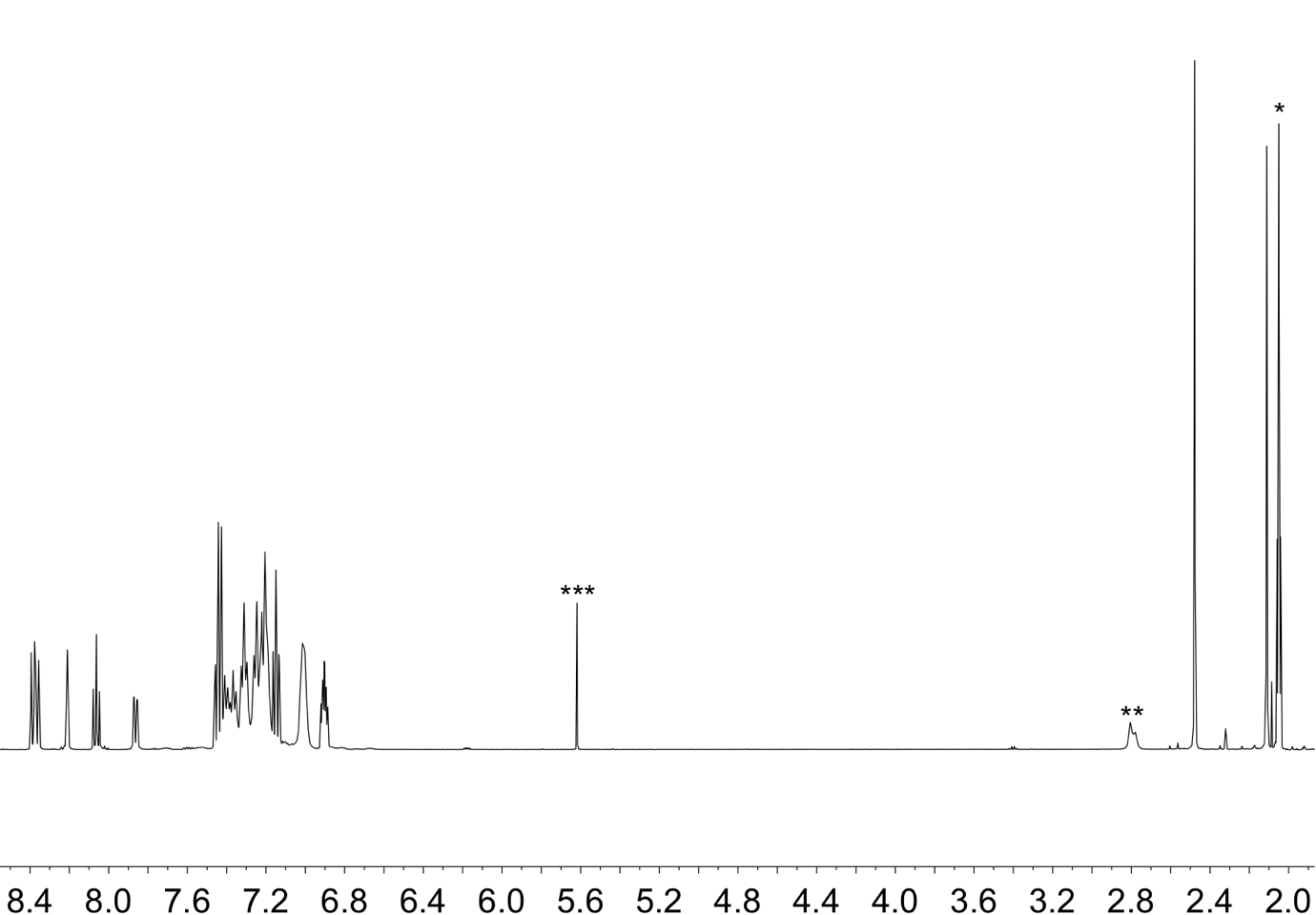


Figure S3. ^1^H NMR spectrum of [Cu(POP)(5,6'-Me_2_bpy)][PF_6_] in acetone-*d*_6_ (500 MHz, 298 K). * = residual acetone-*d*_5_; ** = H_2_O / HOD; *** = CH_2_Cl_2_.


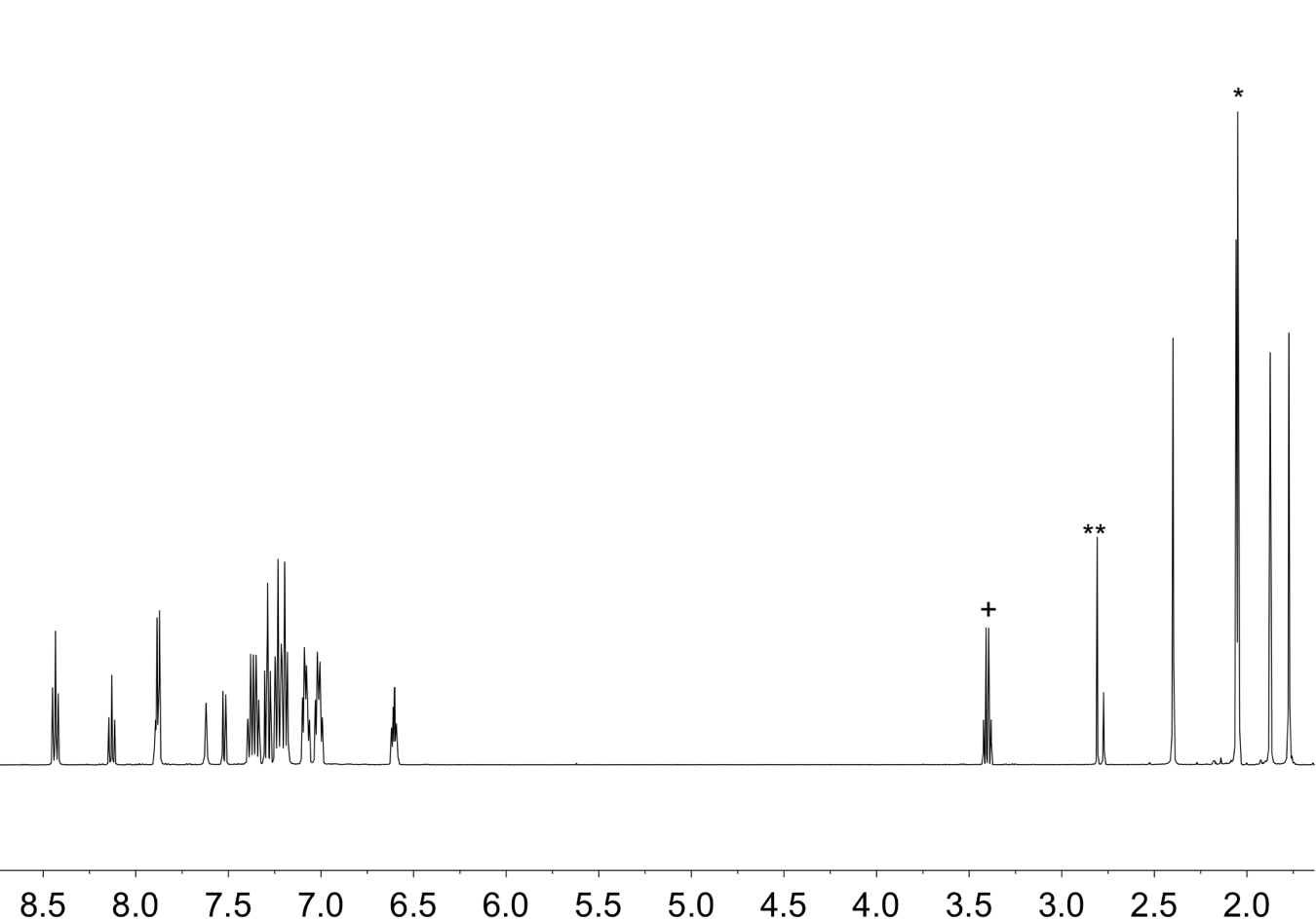


Figure S4. ^1^H NMR spectrum of [Cu(xantphos)(5,6'-Me_2_bpy)][PF_6_] in acetone-*d*_6_ (500 MHz, 298 K). * = residual acetone-*d*_5_ overlapping with the signal for H^Me-A5^; ** = H_2_O / HOD; + = CH_2_ of Et_2_O. Scale: *δ* / ppm.


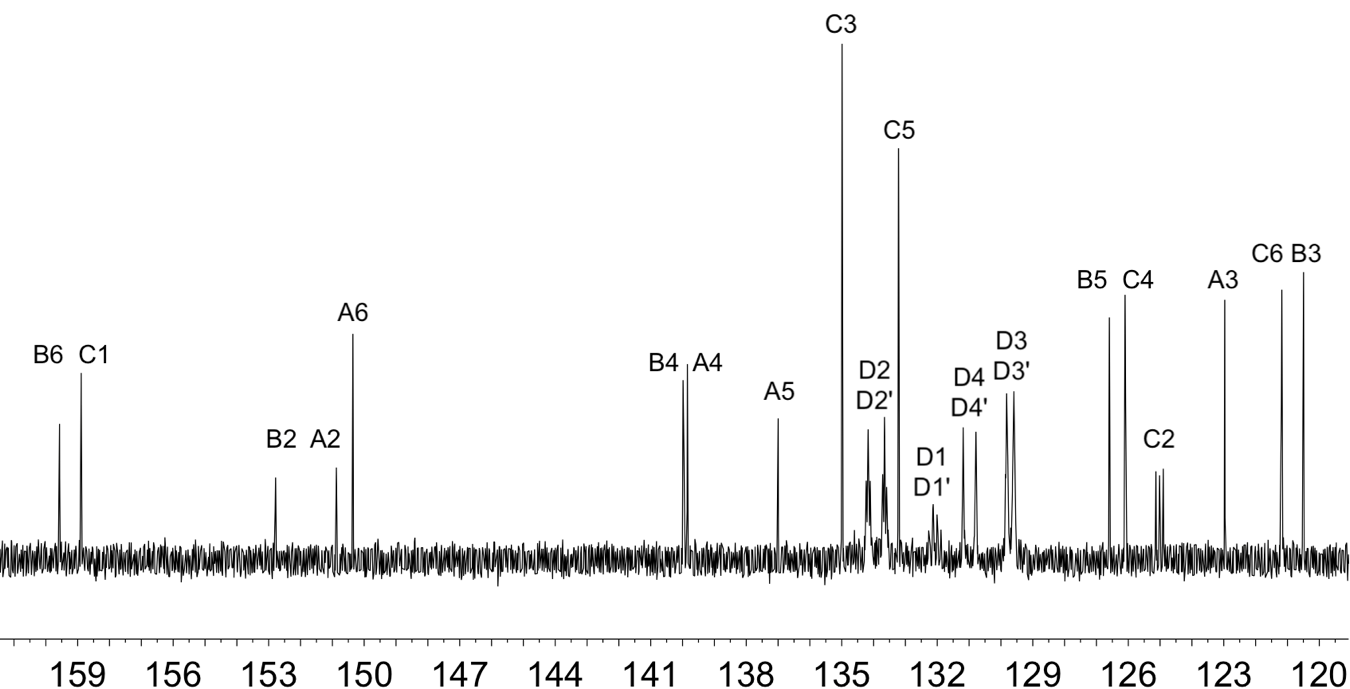


Figure S5. Part of the ^13^C{^1^H} NMR spectrum of [Cu(POP)(5,6'-Me_2_bpy)][PF_6_] in acetone-*d*_6_ (500 MHz, 298 K). Scale: *δ* / ppm.


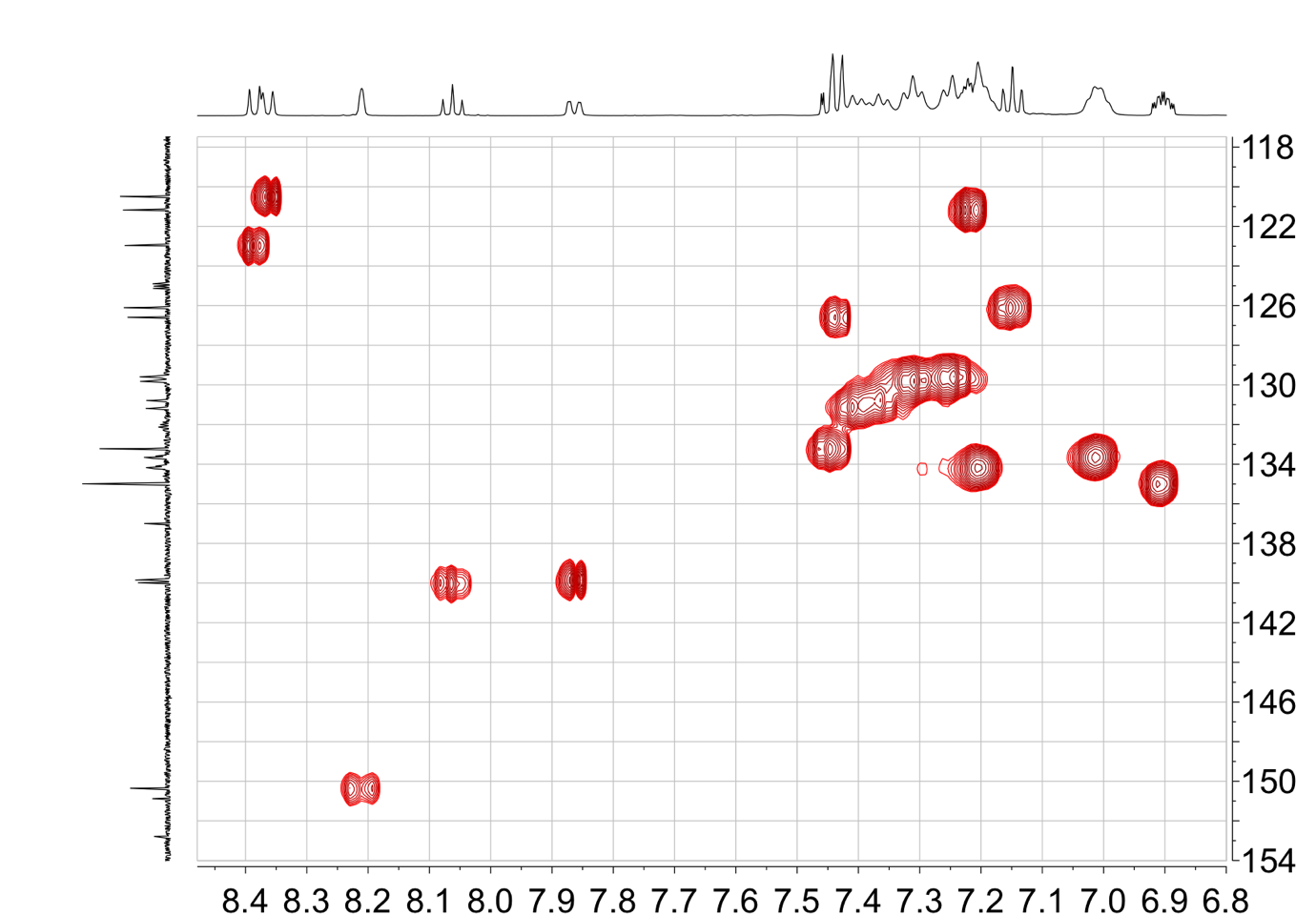


Figure S6. HMQC spectrum of [Cu(POP)(5,6'-Me_2_bpy)][PF_6_] in acetone-*d*_6_ (500 MHz ^1^H, 126 MHz ^13^C, 298 K). Scale: *δ* / ppm.


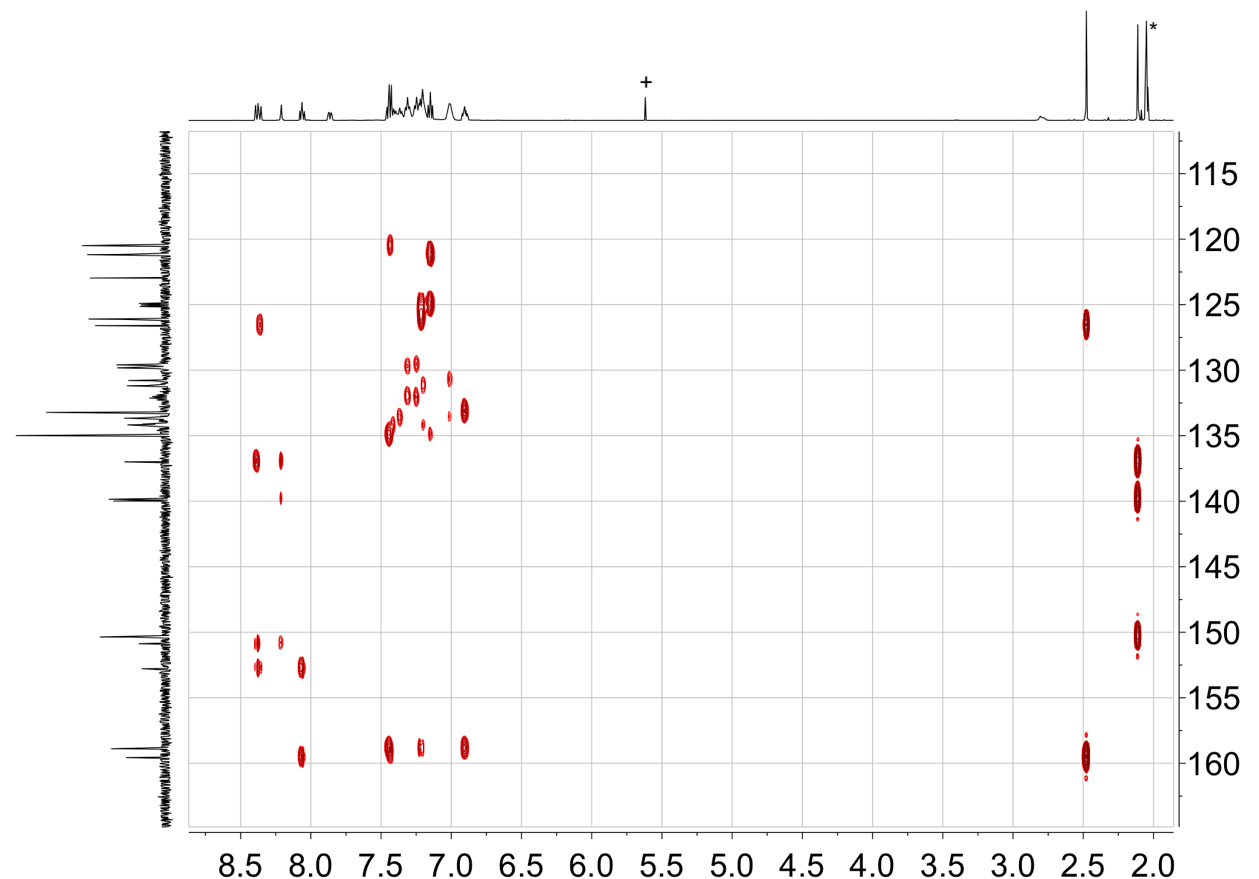


Figure S7. HMBC spectrum of [Cu(POP)(5,6'-Me_2_bpy)][PF_6_] in acetone-*d*_6_ (500 MHz ^1^H, 126 MHz ^13^C, 298 K). * = residual acetone-d_5_; + = CH_2_Cl_2_. Scale: *δ* / ppm.


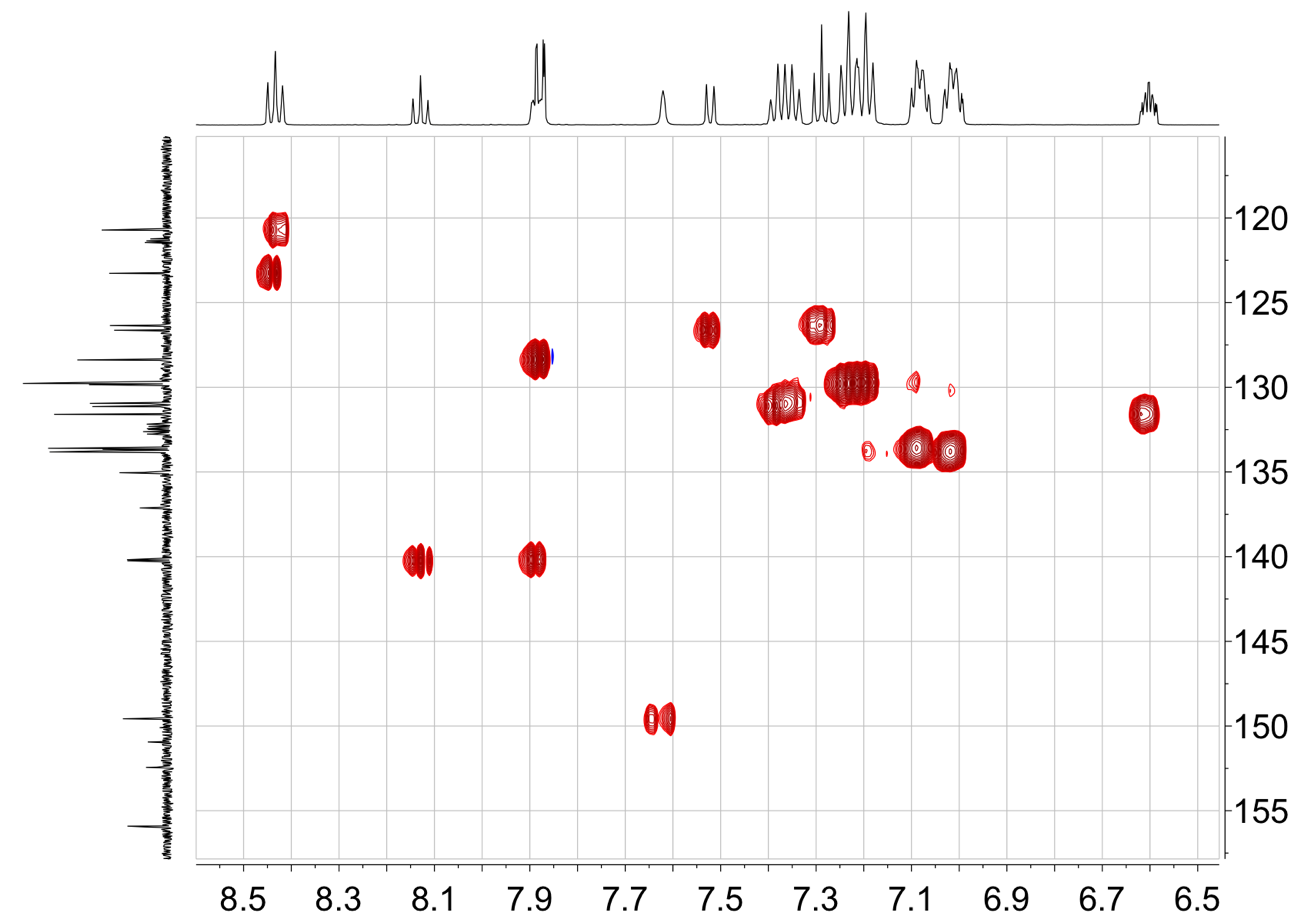


Figure S8. HMQC spectrum of [Cu(xantphos)(5,6'-Me_2_bpy)][PF_6_] in acetone-*d*_6_ (500 MHz ^1^H, 126 MHz ^13^C, 298 K). Scale: *δ* / ppm.


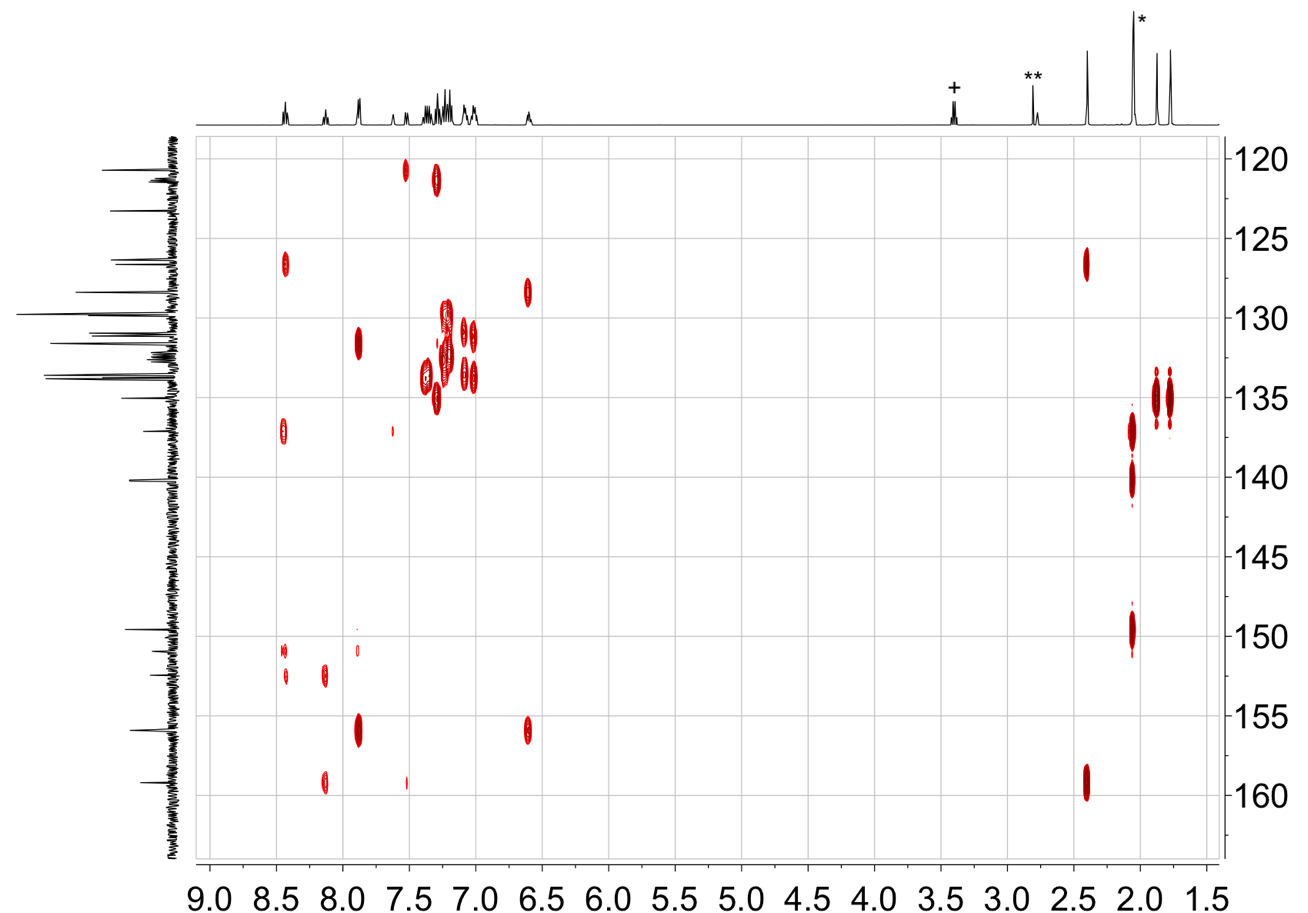


Figure S9. HMBC spectrum of [Cu(xantphos)(5,6'-Me_2_bpy)][PF_6_] in acetone-*d*_6_ (500 MHz ^1^H, 126 MHz ^13^C, 298 K). * = Residual acetone-*d*_5_ overlapping with the signal for H^Me-A5^; ** = H_2_O / HOD; + = CH_2_ of Et_2_O. Scale: *δ* / ppm.


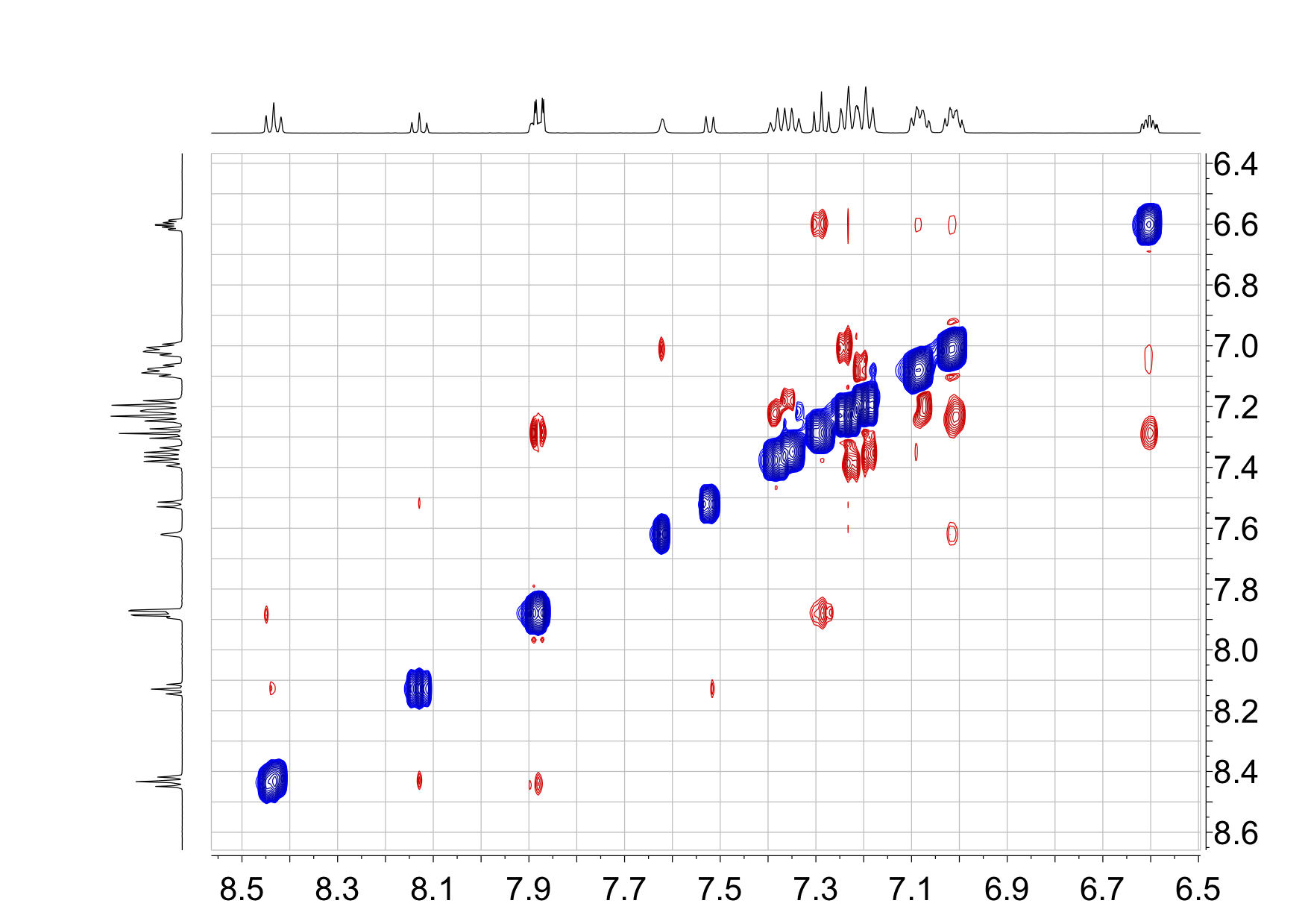


Figure S10. Part of the NOESY spectrum of [Cu(xantphos)(5,6'-Me_2_bpy)][PF_6_] in acetone-*d*_6_ (500 MHz ^1^H, 298 K).

Table S1. Values of *A*_1_, *A*_2_, τ_1_ and τ_2_  for the biexponential fit to the decay using the equation: *τ* = Σ*A_i_τ_i_*/Σ*A_i_*

| **Compound** | ***A*_1_** | **τ_1_** | ***A*_2_** | **τ_2_** | **τ** |
| --- | --- | --- | --- | --- | --- |
| [Cu(POP)(5,6'-Me_2_bpy)][PF_6_] | 0.6612 | 8.236 | 0.2997 | 2.463 | 6.435 |
| [Cu(xantphos)(5,6'-Me_2_bpy)][PF_6_] | 0.614 | 6.384 | 0.3115 | 2.12 | 4.949 |
