# Supplementary material for: Positional Isomerism in the N^N Ligand: How Much Difference Does a Methyl Group Make in [Cu(P^P)(N^N)]+ Complexes?
Source: Molecules. 2020 Jun 15;25(12):2760. doi: 10.3390/molecules25122760 (PMC7356218; doi:10.3390/molecules25122760)

checkCIF/PLATON report

Structure factors have been supplied for datablock(s) brf212_130k

THIS REPORT IS FOR GUIDANCE ONLY. IF USED AS PART OF A REVIEW PROCEDURE FOR PUBLICATION, IT SHOULD NOT REPLACE THE EXPERTISE OF AN EXPERIENCED CRYSTALLOGRAPHIC REFEREE.

No syntax errors found.   CIF dictionary   Interpreting this report

Datablock: brf212_130k

```
Bond precision:  C-C = 0.0023 A          Wavelength=1.34143

Cell:            a=6.4300(2)     b=19.2147(4)       c=8.0931(2)
                 alpha=90        beta=91.147(2)     gamma=90
Temperature:     130 K

                Calculated                 Reported
Volume          999.71(4)                  999.71(4)
Space group     P 21/n                     P 1 21/n 1
Hall group      -P 2yn                     -P 2yn
Moiety formula  C12 H12 N2                 C12 H12 N2
Sum formula     C12 H12 N2                 C12 H12 N2
Mr              184.24                     184.24
Dx,g cm-3       1.224                      1.224
Z               4                          4
Mu (mm-1)       0.365                      0.369
F000            392.0                      392.0
F000’           392.77
h,k,lmax        8,24,10                    8,24,10
Nref            2046                       2030
Tmin,Tmax       0.919,0.975                0.044,1.000
Tmin’           0.902

Correction method= # Reported T Limits: Tmin=0.044 Tmax=1.000
AbsCorr = MULTI-SCAN

Data completeness= 0.992          Theta(max)= 57.094

R(reflections)= 0.0564( 1952)     wR2(reflections)= 0.1698( 2030)

S = 1.190                  Npar= 130
```

The following ALERTS were generated. Each ALERT has the format
test-name_ALERT_alert-type_alert-level.
Click on the hyperlinks for more details of the test.

Alert level B

```
PLAT934_ALERT_3_B Number of (Iobs-Icalc)/SigmaW > 10 Outliers ....          2 Check
```

Alert level C

```
PLAT906_ALERT_3_C Large K Value in the Analysis of Variance ......      3.502 Check
PLAT906_ALERT_3_C Large K Value in the Analysis of Variance ......      2.323 Check
PLAT918_ALERT_3_C Reflection(s) with I(obs) much Smaller I(calc) .          3 Check
PLAT939_ALERT_3_C Large Value of Not (SHELXL) Weight Optimized S .      16.24 Check
```

Alert level G

```
ABSMU01_ALERT_1_G  Calculation of _exptl_absorpt_correction_mu
                not performed for this radiation type.
PLAT912_ALERT_4_G Missing # of FCF Reflections Above STh/L=  0.600         17 Note
PLAT978_ALERT_2_G Number C-C Bonds with Positive Residual Density.          4 Info
PLAT984_ALERT_1_G The  C-f'=    0.0148 Deviates from the B&C-Value     0.0137 Check
PLAT984_ALERT_1_G The  N-f'=    0.0253 Deviates from the B&C-Value     0.0241 Check
```

```
   0 ALERT level A = Most likely a serious problem - resolve or explain
   1 ALERT level B = A potentially serious problem, consider carefully
   4 ALERT level C = Check. Ensure it is not caused by an omission or oversight
   5 ALERT level G = General information/check it is not something unexpected

   3 ALERT type 1 CIF construction/syntax error, inconsistent or missing data
   1 ALERT type 2 Indicator that the structure model may be wrong or deficient
   5 ALERT type 3 Indicator that the structure quality may be low
   1 ALERT type 4 Improvement, methodology, query or suggestion
   0 ALERT type 5 Informative message, check
```

Validation response form

Please find below a validation response form (VRF) that can be filled in and pasted into your CIF.

```
# start Validation Reply Form
_vrf_PLAT906_brf212_130k
;
PROBLEM: Large K Value in the Analysis of Variance ......      3.502 Check
RESPONSE: ...
;
_vrf_PLAT918_brf212_130k
;
PROBLEM: Reflection(s) with I(obs) much Smaller I(calc) .          3 Check
RESPONSE: ...
;
_vrf_PLAT939_brf212_130k
;
PROBLEM: Large Value of Not (SHELXL) Weight Optimized S .      16.24 Check
RESPONSE: ...
;
# end Validation Reply Form
```

It is advisable to attempt to resolve as many as possible of the alerts in all categories. Often the minor alerts point to easily fixed oversights, errors and omissions in your CIF or refinement strategy, so attention to these fine details can be worthwhile. In order to resolve some of the more serious problems it may be necessary to carry out additional measurements or structure refinements. However, the purpose of your study may justify the reported deviations and the more serious of these should normally be commented upon in the discussion or experimental section of a paper or in the "special_details" fields of the CIF. checkCIF was carefully designed to identify outliers and unusual parameters, but every test has its limitations and alerts that are not important in a particular case may appear. Conversely, the absence of alerts does not guarantee there are no aspects of the results needing attention. It is up to the individual to critically assess their own results and, if necessary, seek expert advice.

Publication of your CIF in IUCr journals

A basic structural check has been run on your CIF. These basic checks will be run on all CIFs submitted for publication in IUCr journals (*Acta Crystallographica*, *Journal of Applied Crystallography*, *Journal of Synchrotron Radiation*); however, if you intend to submit to *Acta Crystallographica Section C* or *E* or *IUCrData*, you should make sure that full publication checks are run on the final version of your CIF prior to submission.

Publication of your CIF in other journals

Please refer to the *Notes for Authors* of the relevant journal for any special instructions relating to CIF submission.

PLATON version of 06/01/2019; check.def file version of 19/12/2018

Datablock brf212_130k - ellipsoid plot

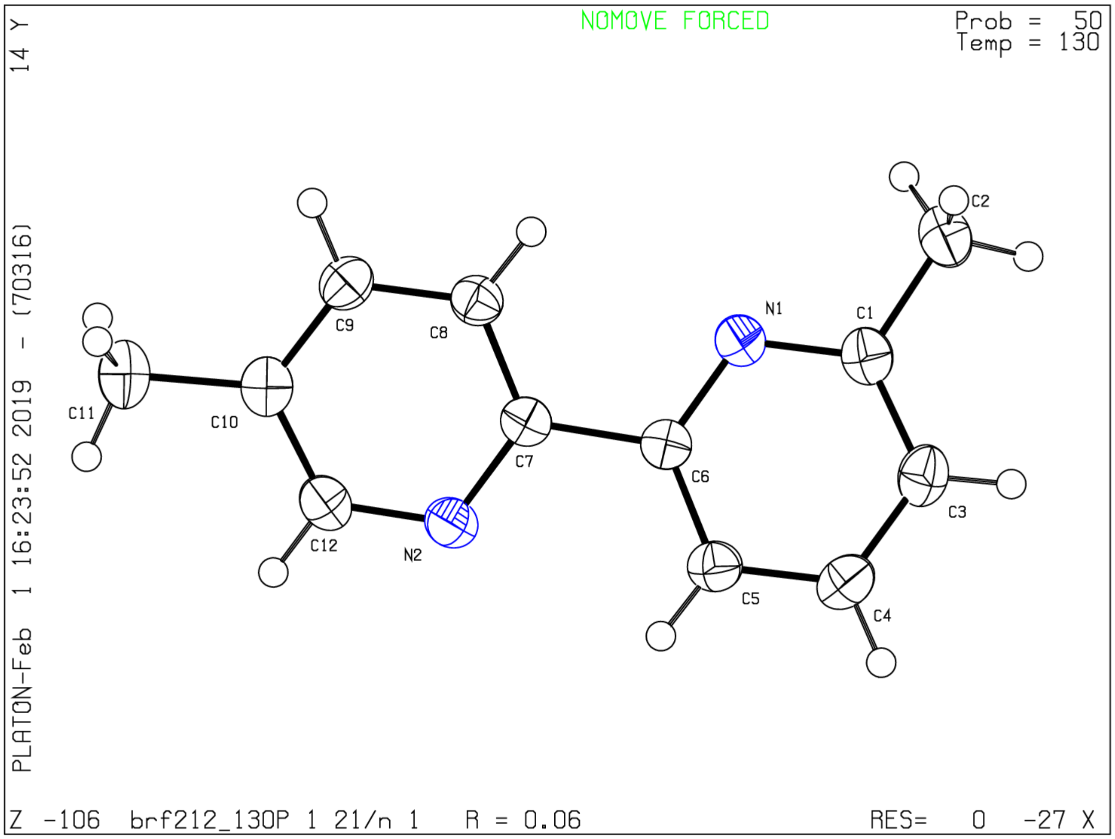

Supplement: Supplementary file 1 [file molecules-25-02760-s001.zip › BRF212checkcif.pdf]
