# Supplementary material for: Positional Isomerism in the N^N Ligand: How Much Difference Does a Methyl Group Make in [Cu(P^P)(N^N)]+ Complexes?
Source: Molecules. 2020 Jun 15;25(12):2760. doi: 10.3390/molecules25122760 (PMC7356218; doi:10.3390/molecules25122760)

checkCIF/PLATON report

Structure factors have been supplied for datablock(s) BRF213_130K

THIS REPORT IS FOR GUIDANCE ONLY. IF USED AS PART OF A REVIEW PROCEDURE FOR PUBLICATION, IT SHOULD NOT REPLACE THE EXPERTISE OF AN EXPERIENCED CRYSTALLOGRAPHIC REFEREE.

No syntax errors found. CIF dictionary Interpreting this report

Datablock: BRF213_130K

Bond precision: C-C = 0.0038 A Wavelength=1.54178

Cell:	a=9.5613(9)	b=14.8515(13)	c=32.472(3)
	alpha=90	beta=90.338(4)	gamma=90
Temperature:	130 K		

	Calculated	Reported
Volume	4610.9(7)	4611.0(7)
Space group	P 21/c	P 1 21/c 1
Hall group	-P 2ybc	-P 2ybc
Moiety formula	C48 H40 Cu N2 O P2, F6 P, C3 H6 O	C51 H46 Cu1 F6 N2 O2 P3
Sum formula	C51 H46 Cu F6 N2 O2 P3	C51 H46 Cu1 F6 N2 O2 P3
Mr	989.36	989.39
Dx,g cm-3	1.425	1.425
Z	4	4
Mu (mm-1)	2.227	2.227
F000	2040.0	2040.0
F000’	2041.50	
h,k,lmax	11,17,39	11,17,38
Nref	8571	8211
Tmin,Tmax	0.745,0.800	0.639,0.753
Tmin’	0.536	

Correction method= # Reported T Limits: Tmin=0.639 Tmax=0.753
AbsCorr = MULTI-SCAN

Data completeness= 0.958 Theta(max)= 68.966

R(reflections)= 0.0392(7611) wR2(reflections)= 0.0536(8211)

S = 0.997 Npar= 586

The following ALERTS were generated. Each ALERT has the format
test-name_ALERT_alert-type_alert-level.
Click on the hyperlinks for more details of the test.

Alert level C

```
PLAT029_ALERT_3_C _diffrn_measured_fraction_theta_full value Low .      0.975 Why?
PLAT911_ALERT_3_C Missing FCF Refl Between Thmin & STh/L=    0.600        157 Report
```

Alert level G

```
PLAT042_ALERT_1_G Calc. and Reported MoietyFormula Strings  Differ     Please Check
PLAT244_ALERT_4_G Low    'Solvent' Ueq as Compared to Neighbors of         P3 Check
PLAT380_ALERT_4_G Incorrectly? Oriented X(sp2)-Methyl Moiety .....        C47 Check
PLAT808_ALERT_5_G No Parseable SHELXL Style Weighting Scheme Found     Please Check
PLAT910_ALERT_3_G Missing # of FCF Reflection(s) Below Theta(Min).          2 Note
PLAT912_ALERT_4_G Missing # of FCF Reflections Above STh/L=  0.600        199 Note
PLAT929_ALERT_5_G No Weight Pars,Obs and Calc R1,wR2,S not Checked          ! Info
PLAT960_ALERT_3_G Number of Intensities with I  <   - 2*sig(I) ...          4 Check
```

---

0 **ALERT level A** = Most likely a serious problem - resolve or explain
0 **ALERT level B** = A potentially serious problem, consider carefully
2 **ALERT level C** = Check. Ensure it is not caused by an omission or oversight
8 **ALERT level G** = General information/check it is not something unexpected

1 ALERT type 1 CIF construction/syntax error, inconsistent or missing data
0 ALERT type 2 Indicator that the structure model may be wrong or deficient
4 ALERT type 3 Indicator that the structure quality may be low
3 ALERT type 4 Improvement, methodology, query or suggestion
2 ALERT type 5 Informative message, check

Validation response form

Please find below a validation response form (VRF) that can be filled in and pasted into your CIF.

```
# start Validation Reply Form
_vrf_PLAT029_BRF213_130K
;
PROBLEM: _diffrn_measured_fraction_theta_full value Low .      0.975 Why?
RESPONSE: ...
;
_vrf_PLAT911_BRF213_130K
;
PROBLEM: Missing FCF Refl Between Thmin & STh/L=    0.600        157 Report
RESPONSE: ...
;
# end Validation Reply Form
```

It is advisable to attempt to resolve as many as possible of the alerts in all categories. Often the minor alerts point to easily fixed oversights, errors and omissions in your CIF or refinement strategy, so attention to these fine details can be worthwhile. In order to resolve some of the more serious problems it may be necessary to carry out additional measurements or structure refinements. However, the purpose of your study may justify the reported deviations and the more serious of these should normally be commented upon in the discussion or experimental section of a paper or in the "special_details" fields of the CIF. checkCIF was carefully designed to identify outliers and unusual parameters, but every test has its limitations and alerts that are not important in a particular case may appear. Conversely, the absence of alerts does not guarantee there are no aspects of the results needing attention. It is up to the individual to critically assess their own results and, if necessary, seek expert advice.

Publication of your CIF in IUCr journals

A basic structural check has been run on your CIF. These basic checks will be run on all CIFs submitted for publication in IUCr journals (*Acta Crystallographica*, *Journal of Applied Crystallography*, *Journal of Synchrotron Radiation*); however, if you intend to submit to *Acta Crystallographica Section C* or *E* or *IUCrData*, you should make sure that full publication checks are run on the final version of your CIF prior to submission.

Publication of your CIF in other journals

Please refer to the *Notes for Authors* of the relevant journal for any special instructions relating to CIF submission.

PLATON version of 06/01/2019; check.def file version of 19/12/2018

Datablock BRF213_130K - ellipsoid plot

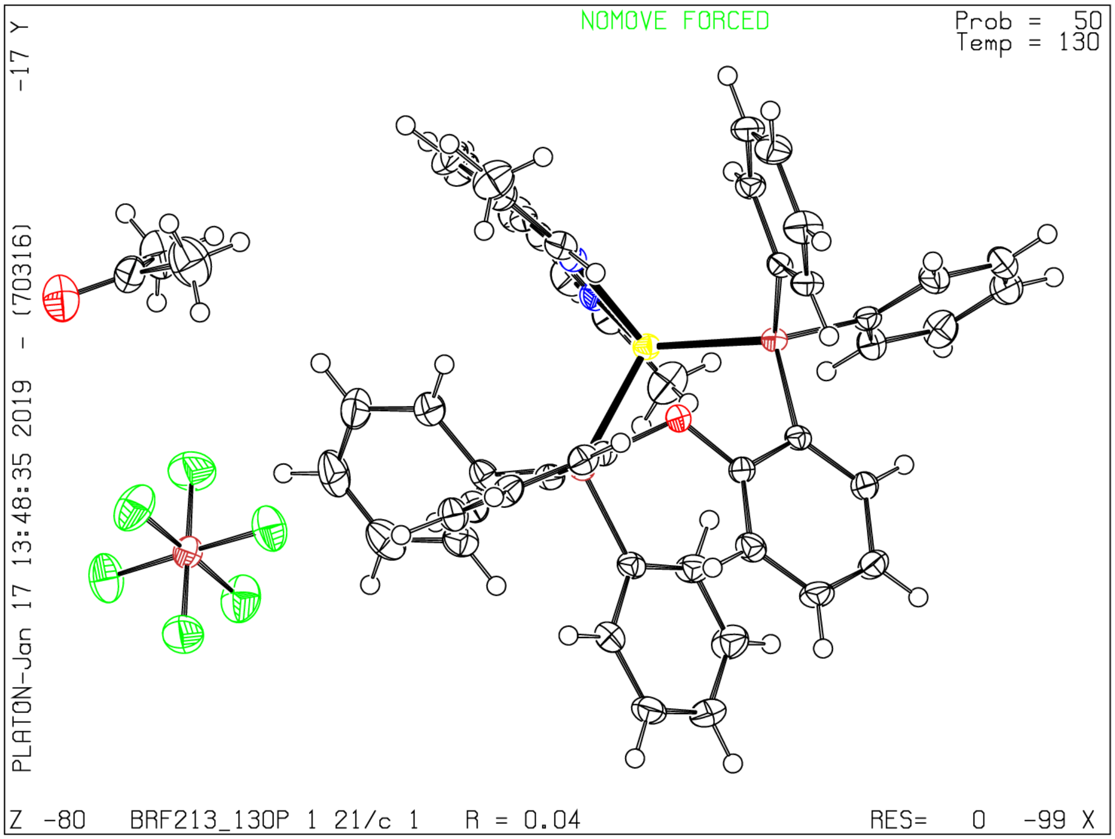

Supplement: Supplementary file 1 [file molecules-25-02760-s001.zip › BRF213checkcif.pdf]
