# Supplementary material for: Positional Isomerism in the N^N Ligand: How Much Difference Does a Methyl Group Make in [Cu(P^P)(N^N)]+ Complexes?
Source: Molecules. 2020 Jun 15;25(12):2760. doi: 10.3390/molecules25122760 (PMC7356218; doi:10.3390/molecules25122760)

checkCIF/PLATON report

Structure factors have been supplied for datablock(s) brf214_130k_0m

THIS REPORT IS FOR GUIDANCE ONLY. IF USED AS PART OF A REVIEW PROCEDURE FOR PUBLICATION, IT SHOULD NOT REPLACE THE EXPERTISE OF AN EXPERIENCED CRYSTALLOGRAPHIC REFEREE.

No syntax errors found.   CIF dictionary   Interpreting this report

Datablock: brf214_130k_0m

Bond precision: C-C = 0.0047 A   Wavelength=1.54178

Cell:	a=17.6502(15)	b=16.8504(15)	c=18.4582(16)
	alpha=90	beta=104.685(3)	gamma=90
Temperature:	130 K		

	Calculated	Reported
Volume	5310.4(8)	5310.4(8)
Space group	P 21/n	P 1 21/n 1
Hall group	-P 2yn	-P 2yn
Moiety formula	2(C51 H44 Cu N2 O P2), 2(F6 P), C H2 Cl2 [+ solvent]	C51 H44 Cu N2 O P2, F6 P, 0.5(Ch2Cl2), 0.75[C4H10O1]
Sum formula	C103 H90 Cl2 Cu2 F12 N4 O2 P6 [+ solvent]	C54.50 H52.50 Cl Cu F6 N2 O1.75 P3
Mr	2027.61	1069.38
Dx,g cm-3	1.268	1.338
Z	2	4
Mu (mm-1)	2.384	2.422
F000	2084.0	2210.0
F000'	2086.81	
h,k,lmax	21,20,22	21,20,21
Nref	9897	9603
Tmin,Tmax	0.559,0.695	0.611,0.753
Tmin'	0.507	

Correction method= # Reported T Limits: Tmin=0.611 Tmax=0.753
AbsCorr = MULTI SCAN

Data completeness= 0.970   Theta(max)= 69.064

R(reflections)= 0.0544(8839)   wR2(reflections)= 0.1768(9603)

S = 1.025   Npar= 608

The following ALERTS were generated. Each ALERT has the format
test-name_ALERT_alert-type_alert-level.
Click on the hyperlinks for more details of the test.

Alert level B

```
PLAT934_ALERT_3_B Number of (Iobs-Icalc)/SigmaW > 10 Outliers ....          4 Check
```

Alert level C

```
PLAT094_ALERT_2_C Ratio of Maximum / Minimum Residual Density ....       2.53 Report
PLAT260_ALERT_2_C Large Average Ueq of Residue Including       Cl1      0.132 Check
PLAT911_ALERT_3_C Missing FCF Refl Between Thmin & STh/L=    0.600        100 Report
PLAT918_ALERT_3_C Reflection(s) with I(obs) much Smaller I(calc) .          4 Check
```

Alert level G

```
FORMU01_ALERT_2_G  There is a discrepancy between the atom counts in the
            _chemical_formula_sum and the formula from the _atom_site* data.
            Atom count from _chemical_formula_sum:C54.5 H52.5 Cl1 Cu1 F6 N2 O1.75
            Atom count from the _atom_site data:  C51.5 H45 Cl1 Cu1 F6 N2 O1 P3
CELLZ01_ALERT_1_G Difference between formula and atom_site contents detected.
CELLZ01_ALERT_1_G ALERT: Large difference may be due to a
            symmetry error - see SYMMG tests
           From the CIF: _cell_formula_units_Z    4
           From the CIF: _chemical_formula_sum  C54.50 H52.50 Cl Cu F6 N2 O1.75 P3
           TEST: Compare cell contents of formula and atom_site data
```

atom	Z*formula	cif sites	diff
C	218.00	206.00	12.00
H	210.00	180.00	30.00
Cl	4.00	4.00	0.00
Cu	4.00	4.00	0.00
F	24.00	24.00	0.00
N	8.00	8.00	0.00
O	7.00	4.00	3.00
P	12.00	12.00	0.00

```
PLAT003_ALERT_2_G Number of Uiso or Uij Restrained non-H Atoms ...          3 Report
PLAT041_ALERT_1_G Calc. and Reported SumFormula    Strings  Differ     Please Check
PLAT042_ALERT_1_G Calc. and Reported MoietyFormula Strings  Differ     Please Check
PLAT045_ALERT_1_G Calculated and Reported Z Differ by a Factor ...       0.50 Check
PLAT051_ALERT_1_G Mu(calc) and Mu(CIF) Ratio Differs from 1.0 by .       1.58 %
PLAT068_ALERT_1_G Reported F000 Differs from Calcd (or Missing)...     Please Check
PLAT072_ALERT_2_G SHELXL First  Parameter in WGHT  Unusually Large       0.11 Report
PLAT083_ALERT_2_G SHELXL Second Parameter in WGHT  Unusually Large       7.01 Why ?
PLAT186_ALERT_4_G The CIF-Embedded .res File Contains ISOR Records          1 Report
PLAT244_ALERT_4_G Low    'Solvent' Ueq as Compared to Neighbors of         P3 Check
PLAT300_ALERT_4_G Atom Site Occupancy of Cl1        Constrained at        0.5 Check
PLAT300_ALERT_4_G Atom Site Occupancy of Cl2        Constrained at        0.5 Check
PLAT300_ALERT_4_G Atom Site Occupancy of C52        Constrained at        0.5 Check
PLAT300_ALERT_4_G Atom Site Occupancy of H52A       Constrained at        0.5 Check
PLAT300_ALERT_4_G Atom Site Occupancy of H52B       Constrained at        0.5 Check
PLAT302_ALERT_4_G Anion/Solvent/Minor-Residue Disorder (Resd  3  )       100% Note
PLAT304_ALERT_4_G Non-Integer Number of Atoms in ...... Resd  3          2.50 Check
PLAT432_ALERT_2_G Short Inter X...Y Contact  Cl1      ..C30              3.11 Ang.
                                                      x,y,z  =      1_555 Check
PLAT605_ALERT_4_G Largest Solvent Accessible VOID in the Structure        322 A**3
PLAT860_ALERT_3_G Number of Least-Squares Restraints ............          18 Note
PLAT868_ALERT_4_G ALERTS Due to the Use of _smtbx_masks Suppressed          ! Info
PLAT910_ALERT_3_G Missing # of FCF Reflection(s) Below Theta(Min).          1 Note
```

```
PLAT912_ALERT_4_G Missing # of FCF Reflections Above STh/L=  0.600        173 Note
PLAT978_ALERT_2_G Number C-C Bonds with Positive Residual Density.          2 Info
```

```
   0 ALERT level A = Most likely a serious problem - resolve or explain
   1 ALERT level B = A potentially serious problem, consider carefully
   4 ALERT level C = Check. Ensure it is not caused by an omission or oversight
  27 ALERT level G = General information/check it is not something unexpected

   7 ALERT type 1 CIF construction/syntax error, inconsistent or missing data
   8 ALERT type 2 Indicator that the structure model may be wrong or deficient
   5 ALERT type 3 Indicator that the structure quality may be low
  12 ALERT type 4 Improvement, methodology, query or suggestion
   0 ALERT type 5 Informative message, check
```

Validation response form

Please find below a validation response form (VRF) that can be filled in and pasted into your CIF.

```
# start Validation Reply Form
_vrf_PLAT094_brf214_130k_0m
;
PROBLEM: Ratio of Maximum / Minimum Residual Density ....       2.53 Report
RESPONSE: ...
;
_vrf_PLAT260_brf214_130k_0m
;
PROBLEM: Large Average Ueq of Residue Including       Cl1      0.132 Check
RESPONSE: ...
;
_vrf_PLAT911_brf214_130k_0m
;
PROBLEM: Missing FCF Refl Between Thmin & STh/L=    0.600        100 Report
RESPONSE: ...
;
_vrf_PLAT918_brf214_130k_0m
;
PROBLEM: Reflection(s) with I(obs) much Smaller I(calc) .          4 Check
RESPONSE: ...
;
# end Validation Reply Form
```

It is advisable to attempt to resolve as many as possible of the alerts in all categories. Often the minor alerts point to easily fixed oversights, errors and omissions in your CIF or refinement strategy, so attention to these fine details can be worthwhile. In order to resolve some of the more serious problems it may be necessary to carry out additional measurements or structure refinements. However, the purpose of your study may justify the reported deviations and the more serious of these should normally be commented upon in the discussion or experimental section of a paper or in the "special_details" fields of the CIF. checkCIF was carefully designed to identify outliers and unusual parameters, but every test has its limitations and alerts that are not important in a particular case may appear. Conversely, the absence of alerts does not guarantee there are no aspects of the results needing attention. It is up to the individual to critically assess their own results and, if necessary, seek expert advice.

Publication of your CIF in IUCr journals

A basic structural check has been run on your CIF. These basic checks will be run on all CIFs submitted for publication in IUCr journals (*Acta Crystallographica*, *Journal of Applied Crystallography*, *Journal of Synchrotron Radiation*); however, if you intend to submit to *Acta Crystallographica Section C* or *E* or *IUCrData*, you should make sure that full publication checks are run on the final version of your CIF prior to submission.

Publication of your CIF in other journals

Please refer to the *Notes for Authors* of the relevant journal for any special instructions relating to CIF submission.

PLATON version of 06/01/2019; check.def file version of 19/12/2018

Datablock brf214_130k_0m - ellipsoid plot

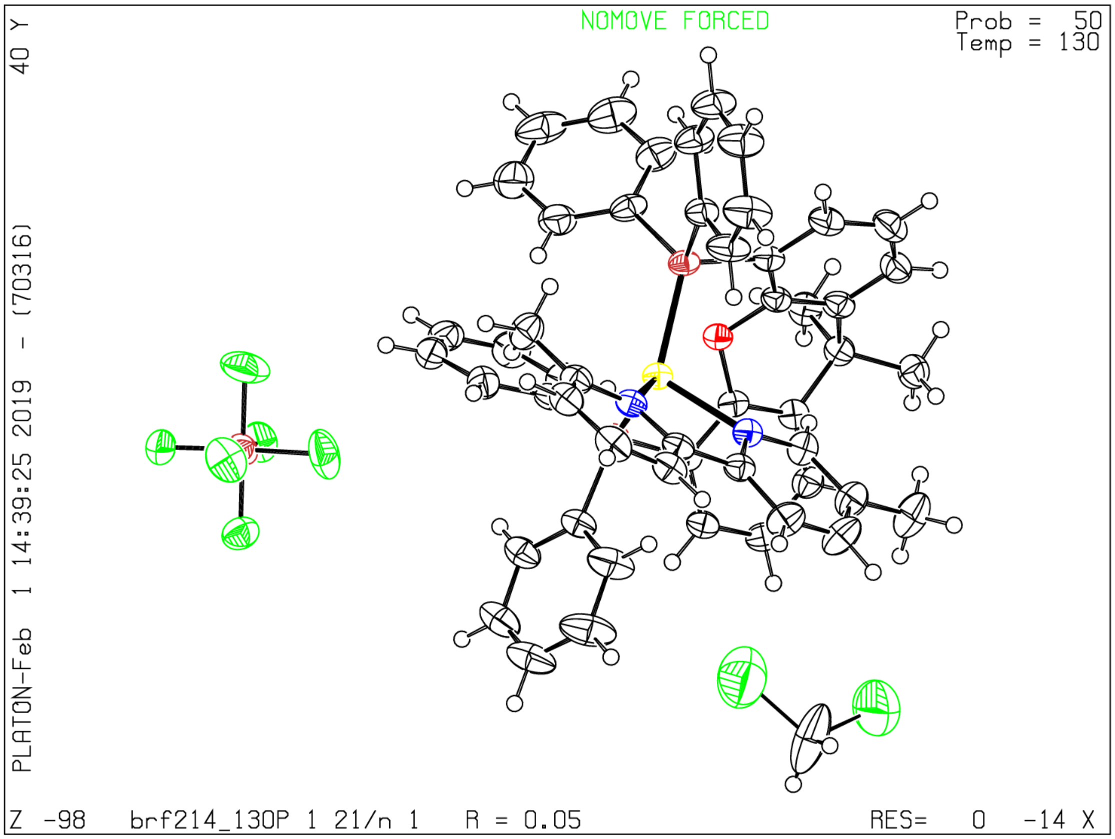

Supplement: Supplementary file 1 [file molecules-25-02760-s001.zip › BRF214checkcif.pdf]
